# A single mutation in *rapP* induces cheating to prevent cheating in *Bacillus subtilis* by minimizing public good production

**DOI:** 10.1038/s42003-018-0136-1

**Published:** 2018-09-04

**Authors:** Nicholas A. Lyons, Roberto Kolter

**Affiliations:** Department of Microbiology and Immunobiology, Harvard Medical School, Boston, MA 02115 USA

## Abstract

Cooperation is beneficial to group behaviors like multicellularity, but is vulnerable to exploitation by cheaters. Here we analyze mechanisms that protect against exploitation of extracellular surfactin in swarms of *Bacillus subtilis*. Unexpectedly, the reference strain NCIB 3610 displays inherent resistance to surfactin-non-producing cheaters, while a different wild isolate is susceptible. We trace this interstrain difference down to a single amino acid change in the plasmid-borne regulator RapP, which is necessary and sufficient for cheater mitigation. This allele, prevalent in many *Bacillus* species, optimizes transcription of the surfactin operon to the minimum needed for full cooperation. When combined with a strain lacking *rapP*, NCIB 3610 acts as a cheater itself—except it does not harm the population at high proportions since it still produces enough surfactin. This strategy of minimal production is thus a doubly advantageous mechanism to limit exploitation of public goods, and is readily evolved from existing regulatory networks.

## Abstract (web summary)

Lyons and Kolter describe a single-point mutation in the plasmid-borne gene *rapP* of *Bacillus subtilis* that optimizes surfactin transcription to express the minimum required for cooperation. The decrease in the production of this public good significantly prevented the exploitation of cooperative traits by cheaters.

## Introduction

Cooperative systems, in which some members of a population help others at their own cost, are ubiquitous in nature yet can be vulnerable to exploitation by non-cooperative individuals that partake of the benefit without paying the cost. These exploitative cheaters cannot thrive on their own but can invade a community with a negative frequency-dependent fitness advantage over the cooperators that is detrimental to the overall population—two of the hallmarks of cheating. Some of the most studied cheating situations are in microbial species since they exhibit many cooperative behaviors, including secreted molecules like siderophores and surfactants, that are often expressed in multicellular contexts like fruiting body formation, swarms, and biofilms^1,2^.

Because cooperative systems are so pervasive, mechanisms to prevent cheating must be as prevalent. Identified mechanisms tend to fall into a handful of different strategies: restrict cooperation to genetic relatives usually via kin discrimination or population bottlenecks^3–15^, only engage in the cooperative trait when it is not rate-limiting^16–21^, limit how public the good actually is^22–26^, couple the cooperative act with other important behaviors such as intracellular metabolism or antimicrobial resistance so defection is more costly^27–42^, continually diversify the shared molecules such that other alleles cannot use them^27,43–46^, or spatially structure the population so producers are more likely to be surrounded by other cooperators^47–58^. However, most of these studies were done with a limited diversity of microbial species and cooperative traits (usually *Pseudomonas aeruginosa* quorum sensing or iron acquisition), and thus may not be representative of all the evolved mechanisms out there. Additionally, experiments are typically performed using a single strain of a given species, so it is not known whether the identified cheater control mechanisms are conserved or if other strains use different mechanisms.

In this study we took advantage of a different cooperative multicellular system: swarming in *Bacillus subtilis*, which is absolutely dependent on the secreted molecule surfactin^59^. We previously showed that *B. subtilis* uses an antagonistic kin discrimination system to prevent this public good from being stolen by unrelated strains^13,60,61^, but this system would not protect against spontaneous cheater mutants that arise from within a kin population. Investigating this scenario, we found that different strains have different responses to the presence of a surfactin-non-producing mutant. We traced this intraspecific difference down to a single mutation in a plasmid-borne gene *rapP* whose protein product regulates major developmental transcriptional cascades. This mutation results in the minimal production of surfactin needed to swarm, thus maintaining the full benefits of cooperation while lowering its cost and exploitability. RapP also effectively turned cells into cheaters, as the minimal-producers had an advantage over the normal-production strains. This strategy represents a novel mechanism to prevent cheating of publically available goods that is straightforwardly evolvable, and may be found more widely in other species.

## Results

### Surfactin cheating in closely related strains

**Fig. 1:**
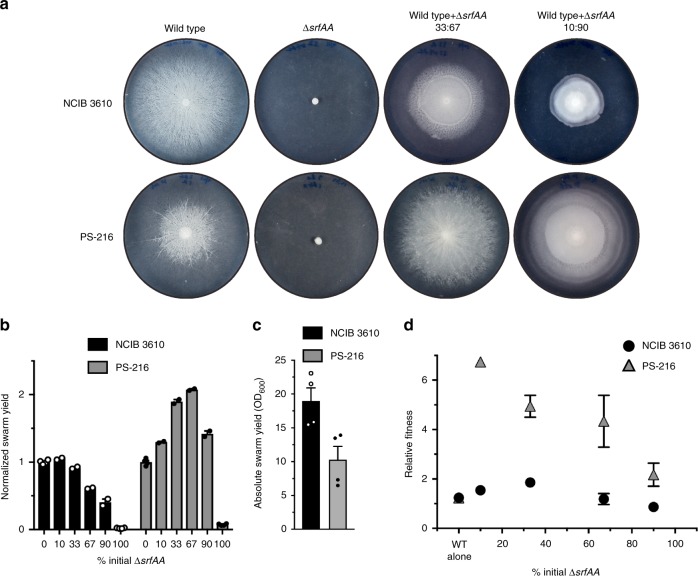
Effect of a non-producer mutant in different *B. subtilis* strain backgrounds. **a** Representative examples of swarm plates of wild type, surfactin mutant (*∆srfAA*), and varying ratios of wild type + ∆*srfAA* mixtures in two closely related strains of *B. subtilis*, NCIB 3610 and PS-216. Plates are 8 cm in diameter. **b** Total cellular yield, as determined by OD_600_ readings of entire swarms, normalized to the value of wild-type alone (0% mutant). Averages of biological replicates (0% and 100% *n* = 2, others *n* = 4) ± standard error of the mean (SEM). The values statistically different from wild-type alone are 67%, 90%, and 100% for NCIB 3610 (*P* = 0.0024, <0.0001, <0.0001) and all for PS-216 (*P* = 0.0197, <0.0001, <0.0001, 0.0011, <0.0001). **c** Mean absolute values of wild-type swarm yields of both strains, in OD_600_ units. *n* = 4, error bars = SEM, *P* = 0.0200. **d** Relative fitness of the *∆srfAA* mutant in swarms with wild type as determined by flow cytometry; each point is the mean ± SEM (wild-type alone *n* = 4, others *n* = 2), some error bars are not visible because they are smaller than the symbol. The wild-type alone values represent the fitness of mixtures of wild-type strains expressing different fluorescent proteins in varying ratios. All PS-216 values were significantly different from wild-type alone (*P* < 0.0001) except 90% mutant (*P* = 0.142), no NCIB 3610 ratios had significant fitness differences

We first tested whether a non-cooperating *B. subtilis* mutant exhibited phenotypes typical of cheating. Cheating has been observed in the standard lab strain NCIB 3610’s biofilm matrix components^57,62,63^ and derived lab strains’ quorum-sensing molecules in swarms^45,64^. We wanted to verify this and compare NCIB 3610 to the closely related strain PS-216 (ref. ^65^), as we previously found a number of differences in cooperative genes among *B. subtilis* strains^61^. PS-216, unlike NCIB 3610 but like most other *B. subtilis* isolates, is a mucoid strain and thus may have different approaches to production of extracellular substances. To assay for cheating behavior, we combined cells harboring a direct deletion of the public good surfactin (*∆srfAA*) with wild-type cells in varying ratios and spotted the mixtures on swarm-inducing media (Fig. 1a). After spreading across the entire plate, swarms were scraped off the agar, OD_600_ readings were taken to determine total cellular yields, and final ratios of wild type: *∆srfAA* were measured by flow cytometry. Initial tests verified that OD_600_ readings tracked very closely with the more direct but laborious method of measuring cell numbers by plating and counting colonies (Supplementary Fig. 1A) and is thus a good indicator of reproductive success of the swarm.

In NCIB 3610, increasing the initial percentage of *∆srfAA* cells caused the total yield of the swarms to decrease (Fig. 1b, black bars), as expected of a cheater harming the population by not producing the public good. However, in PS-216 the number of cells actually increased with added *∆srfAA*, to over two-fold in the 67% *∆srfAA* swarms, then decreased somewhat in the 90% *∆srfAA* swarms (though still higher than wild-type alone) (Fig. 1b, gray bars). This was unexpected, and was accompanied by a change in swarm morphology to more resemble non-mucoid strains like NCIB 3610 (Fig. 1a, bottom right). Also, the improved yields of PS-216 + *∆srfAA* brought it to similar levels as NCIB 3610, which consistently produced denser swarms (Fig. 1c) that expanded at a faster rate (Supplementary Fig. 1B).

When we measured the relative fitness of the mutant in the swarms, we again found a marked difference between strains. This time PS-216 showed the expected cheater phenotype of a negative frequency-dependent fitness advantage of *∆srfAA* over wild type, with low mutant ratios having a fitness over six and high ratios only around two (Fig. 1d, gray triangles). NCIB 3610, though, did not exhibit much of an advantage, peaking at less than two in the 33% *∆srfAA* swarms (Fig. 1d, black circles). The NCIB 3610 strain must therefore have some previously unknown inherent cheater resistance mechanism that limits the fitness advantage non-producers enjoy in other strain backgrounds.

In summary, the PS-216 swarms were helped by the presence of non-producing mutants, but because those mutant cells had a large fitness advantage they would eventually take over the population, leading to loss of the cooperative trait. NCIB 3610, on the other hand, was more sensitive to the addition of non-producers in terms of total swarm yield, but was guarded against their takeover because they have little or no advantage over wild type. So while neither strain showed both of the typical characteristics of being cheated, we consider NCIB 3610 as being cheater-protected because its cooperative swarming should be more evolutionarily stable than PS-216, which we consider cheating-vulnerable. Surfactin is thus an exploitable public good, unlike the surfactant in *P. aeruginosa* rhamnolipids that are public but guarded from exploitation through tight regulation^21^ and *Pseudomonas putida* putisolvin that is neither public nor exploitable^66^.

### Cheater prevention due to a plasmid-borne allele

To see if the difference between the strains was due to the spatial distribution within swarms, which is known to affect cheater phenotypes^47–58^, we examined fluorescently labeled wild type and mutant strains under a stereomicroscope. In both NCIB 3610 and PS-216 we saw even distribution of wild type and *∆srfAA* cells in all areas of the swarms, similar to the wild-type + wild-type control, despite the uneven abundances of each strain (Supplementary Fig. 2A, B). In contrast, *∆hag* cells that do not produce flagella and thus cannot swarm on their own did not spread out much beyond the initial inoculum spot (Supplementary Fig. 2C), indicating the lack of cheating of this private good (flagella). Cheater suppression in NCIB 3610 is thus not due to prevention of *∆srfAA* cells from spreading along with wild-type cells.

**Fig. 2:**
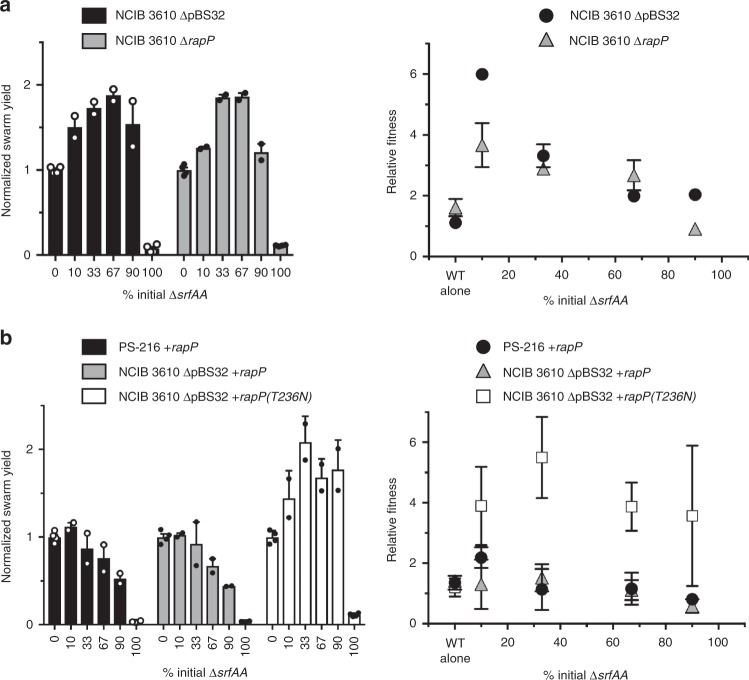
Cheater resistance is lost by removing endogenous plasmid pBS32 or the *rapP* gene on it. **a** *∆srfAA* cheating assays as in Fig. 1, with strains of NCIB 3610 either missing the plasmid (∆pBS32) or with the *rapP* gene interrupted by a transposon insertion (∆*rapP*). Total cell yield of each swarm normalized to the value of wild-type alone (0% mutant); averages of four (0% and 100%) or two replicates (all others). Relative fitness of the *∆srfAA* mutant; each point is the mean ± SEM of independent biological replicates (0% *n* = 4, others *n* = 2). All yields were significantly different from wild-type alone (*P* < 0.0001) except ∆*rapP* 10% and 90% (*P* = 0.0388 and 0.0950); for fitness measurements, 10% and 33% mutant were statistically significant in both ∆pBS32 (*P* < 0.0001 and 0.0009) and ∆*rapP* (*P* = 0.0019 and 0.0477). **b** Cheating assays in which *rapPphrP* or *rapP(T236N)phrP* has been inserted into the chromosome of PS-216 or NCIB 3610 ∆pBS32. Statistically significant swarm yields included PS-216 + *rapP* 90% and 100% (*P* = 0.0003 and <0.0001), ∆pBS32 + *rapP* 67%, 90%, and 100% (*P* = 0.0102, <0.0001, <0.0001), and all ∆pBS32 + *rapP(T236N)* values (10% *P* = 0.0006, others *P* < 0.0001). Neither *rapP* addback had significant fitness values, but all ∆pBS32 + *rapP(T236N)* were significant (*P* < 0.0001 for all but 90% *P* = 0.0004)

We then wondered what the mechanism was behind this inherent cheater mitigation in NCIB 3610, and how come it is missing in PS-216. The genomes of PS-216 and NCIB 3610 are very similar, with only 140 single-nucleotide polymorphisms (SNPs) between them, but three large components are missing in PS-216: the ICEBs1 conjugative element, the SPβ prophage, and the plasmid pBS32 (ref. ^67^). Because previous studies have implicated pBS32 genes in developmental regulation^68–72^, we first tested a strain of NCIB 3610 lacking this plasmid (∆pBS32) in our cheating assay. The phenotype of NCIB 3610 ∆pBS32 resembled PS-216 much more than its parent strain: the swarm yield increased with added ∆*srfAA* cells and the mutant had a clear fitness advantage over the wild-type surfactin producer (Fig. 2a). This suggests the cheater protection phenotype is due to gene(s) on this plasmid.

Plasmid pBS32 has 84,215 bp, 35% GC (lower than the ~42% typical of *B. subtilis*, suggestive of horizontal transfer), around two copies per cell, and 102 genes^69,72^. There was, however, one obvious candidate operon to test first: *rapPphrP*. Rap proteins are regulatory phosphatases that typically remove phosphates from signaling proteins, thus inhibiting them and preventing transcriptional activation of regulons that control things like sporulation, competence, and biofilm formation^73^. Secreted Phr peptides inhibit the cognate Rap in a quorum-dependent fashion, thus allowing expression of the regulons at appropriate cell densities^74^. RapP is unusual is this regard, however, as it does not respond to PhrP inhibition due to a mutation of the conserved asparagine 236 (present in other Rap phosphatases) to threonine^70,71^. RapP therefore acts as a constitutive repressor of its targets Spo0F and ComA that are regulators of many genes necessary for swarming, including surfactin synthesis, and as a result a strain with this allele of *rapP* has a competitive growth advantage in liquid medium^75^.

We therefore tested the effect of deleting just *rapP* and keeping the rest of the plasmid intact. The resulting NCIB 3610 ∆*rapP* strain phenocopied the ∆pBS32 strain in both swarm yield and mutant fitness (Fig. 2a), suggesting *rapP* is the only gene on the plasmid contributing to cheater protection. To confirm this, we performed rescue experiments by inserting the *rapPphrP* operon into a chromosomal locus of the plasmidless strain, creating NCIB 3610 ∆pBS32 + *rapP*. Adding back *rapP* completely mitigated cheating, returning the number of cells and fitness back to wild-type NCIB 3610 levels (Fig. 2b), consistent with RapP being the cause behind the cheater resistance in this strain.

We next wanted to see if *rapPphrP* was sufficient for cheater prevention in addition to being necessary. We thus inserted the operon into the chromosome of PS-216, which is very similar to NCIB 3610 but does contain SNPs in key regulatory genes like *oppD*, *comP*, *degQ*, and *sigH*^67^, and has not co-evolved its transcriptional regulons with this unusual Rap. Despite these differences, the PS-216 + *rapP* strain has an almost identical response to the ∆*srfAA* mutant as NCIB 3610: lower cellular yield but resistance to the mutant’s fitness advantage (Fig. 2b).

As mentioned, RapP is rare among Rap phosphatases in that its N236T mutation confers constitutive repressive activity; otherwise it would behave like any other Rap, of which there are many in every *B. subtilis* strain^76^. We therefore tested whether adding back a non-constitutive version of RapP with residue 236 mutated back to the canonical Asn would also mitigate cheating. It did not, as NCIB 3610 ∆pBS32+*rapP(T236N)* was exploited by the ∆*srfAA* mutant just like the strains without any *rapP*, though it showed much more variability (Fig. 2b). All together, these data indicate that cheating can easily be prevented by a single amino acid change in a single gene.

### Cheater resistance via RapP regulation of public good genes

**Fig. 3:**
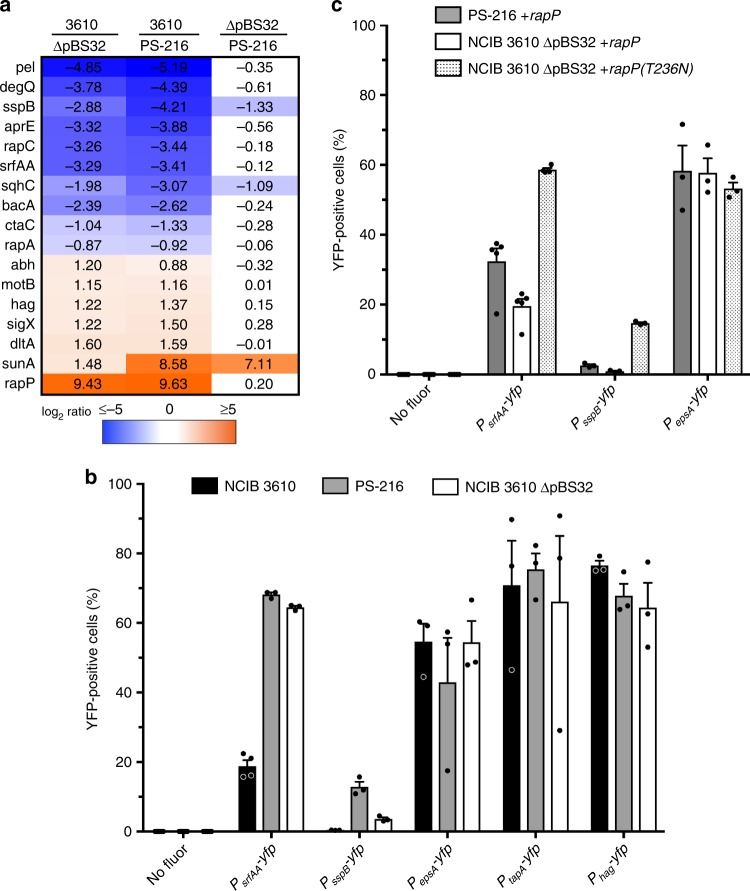
Effect of RapP on gene expression. **a** Heat map of genes with at least two-fold change in expression between swarming strains. Each column is the log_2_ ratio of wild-type NCIB 3610, PS-216, or NCIB 3610 ∆pBS32. Only the expression differences that were statistically significant (*P* < 0.05 by two-tailed *t*-test) are shown; see Supplementary Data 1 for full results of all genes examined. The extreme differences displayed by *sunA* and *rapP*, which are in the SPβ prophage and plasmid pBS32, are due to the absence of the gene in one of the strains. **b** Percentage of swarming cells transcribing *yfp* from the indicated promoters in each strain, as determined by flow cytometry. Controls without *yfp* (no fluor) were used to set thresholds for YFP-positive events for each strain. NCIB 3610 is statistically different from both PS-216 and NCIB 3610 ∆pBS32 reporters for *srfAA* (*P* = 2.44 × 10^−6^ and 3.41 × 10^−6^) and *sspB* (*P* = 1.03 × 10^−3^ and 1.72 × 10^−3^). All are averages of three biological replicates; error bars represent SEM. **c** Transcriptional reporter expression in strains with *rapPphrP* inserted into their chromosomes. NCIB 3610 ∆pBS32+*rapP(T236N)* is significantly different from PS216+*rapP* and NCIB 3610 ∆pBS32+*rapP* in *srfAA* (*P* = 4.93 × 10^−4^ and 7.95 × 10^−7^) and *sspB* (*P* = 1.04 × 10^−5^ and 1.78 × 10^−6^) reporters. All are averages of at least three biological replicates, error bars represent SEM

Given this protein’s known effect on signaling proteins Spo0F and ComA^70,71^, we looked at the expression levels of some of the major targets of these signaling proteins using nanoString nCounter, a probe hybridization-based assay^77^. We examined the transcript levels of 73 genes chosen to represent a cross-section of *B. subtilis* regulons (see Supplementary Data 1 for list of genes). Though it is not a complete global analysis, this technique requires much less sample input and so allowed us to analyze transcriptional dynamics in early swarm development when cell counts are not high. When we compared the mRNA abundances of the three strains of interest—NCIB 3610, PS-216, and NCIB 3610 ∆pBS32—we found significant differences in 17 genes (Fig. 3a). These differences were almost entirely attributable to NCIB 3610, however, as PS-216 and NCIB 3610 ∆pBS32 had nearly identical gene expression. If we exclude *sunA* and *rapP* that are absent in NCIB 3610 ∆pBS32 and/or PS-216, the eight most-different genes are down-regulated in NCIB 3610, and none of the genes induced in NCIB 3610 are more than three-fold higher (Supplementary Data 1). These data support the hypothesis that the phenotypic differences between NCIB 3610 and PS-216 seen in Fig. 1 are largely due to the constitutively repressive activity of RapP on plasmid pBS32.

RapP is thought to repress Spo0F and ComA, and has previously been shown to affect expression of the extracellular matrix operon *eps*, the sporulation gene *spoIIG*, the *srfA* surfactin synthetase operon, and a different response regulator *rapA*^70,71^. Of these, we found *srfAA, rapA*, and the late-sporulation gene *sspB* were indeed different between strains (Fig. 3a). However, *epsA* did not show a significant difference with or without pBS32 in our conditions, nor did *tasA, sinR, slrR, abrB, sdpA*, and *skfB* that are in the same Spo0F–Spo0A transcriptional network^78^ (Supplementary Data 1). This could indicate a different regulatory architecture in swarms than biofilms, or that RapP preferentially interacts with ComA over Spo0F. Consistent with this, all the genes in our dataset from the ComA regulon^79^ were lower in NCIB 3610: *pel, degQ, rapC, srfAA*, and *rapA* (Fig. 3a).

To verify these results and examine expression at a single cell level, we measured fluorescent transcriptional reporters of several candidate genes by flow cytometry: *srfAA*, *sspB*, *epsA, tapA*, and the flagellum gene *hag* (which should be highly expressed in swarming cells but was slightly higher in NCIB 3610 in our nanoString data). The promoter of each was placed in front of the yellow fluorescent protein (*yfp*) gene, and YFP levels in individual swarming cells were compared to a non-fluorescent control to determine the percentage of the population transcribing the promoter. We observed robust expression from promoters of *hag*, *epsA*, and *tapA*, but no difference between strains (Fig. 3b). Expression from the *srfAA* promoter was extremely high in PS-216 and NCIB 3610 ∆pBS32, with 68% and 65% of cells fluorescing yellow, but much lower in NCIB 3610, where less than 19% of cells definitively expressed YFP (Fig. 3b). This is similar to previous results in *B. subtilis* biofilms and sliding populations^80,81^, except that we do not see a separate subpopulation of surfactin-producing cells but rather a slight shift of the entire population (Supplementary Fig. 3A). Expression from *P*_*sspB*_ was also significantly higher in PS-216 and NCIB 3610 ∆pBS32 than NCIB 3610, from 0.356 to 12.9% of cells (Fig. 3b), and all clearly were from a separate subpopulation of sporulating cells (Supplementary Fig. 3C). Since the profiles of the *srfAA* reporter seemed unimodal compared to the *sspB* reporter, we also analyzed the median fluorescence levels of *P*_*srfAA*_*-yfp* strains (Supplementary Fig. 3D). The results were much the same, with NCIB 3610 only having 1.80× the level of the negative control while PS-216 and NCIB 3610 ∆pBS32 were 6.16 and 5.43 times as bright.

Because the profiles of NCIB 3610 ∆pBS32 so closely matched PS-216, we tested the transcriptional reporters in the *rapPphrP* addback strains to see whether RapP was the sole cause of the observed phenotype. As expected, adding *rapP* to PS-216 and NCIB 3610 ∆pBS32 drastically lowered the expression of *P*_*srfAA*_ and *P*_*sspB*_, but had no effect on *P*_*epsA*_ (Fig. 3c). As with the cheating assay above, this effect was dependent on the N236T mutation, since the Asn reversion exhibited elevated transcription of these two reporters.

We next wanted to verify that the ultimate output of *srfAA*—surfactant in the swarm—matched the observed transcriptional differences. We thus performed a droplet collapse assay on swarm supernatants (Supplementary Fig. 4) to approximate the concentration of surfactin in each strain. The results showed a 7- and 15-fold difference in total surfactant between NCIB 3610 and PS-216 or NCIB 3610 ∆pBS32, and 11- and 19-fold when normalized to the number of cells in each swarm (Table 1). This supports the idea that the two cheater-vulnerable strains produce vastly more of the public good relevant to swarming than the resistant strain, which minimizes production.

**Table 1:** Estimation of surfactant concentration by droplet collapse assay

Strain	Total surfactant in swarm (µM)	Surfactant per cell (fM)
NCIB 3610	37 ± 5.4	16 ± 3.3
PS-216	260 ± 48	170 ± 39
NCIB 3610 ∆pBS32	560 ± 60	310 ± 34
NCIB 3610 *∆srfAA*	–	–

Dashes indicate no detectable surfactant activity

**Fig. 4:**
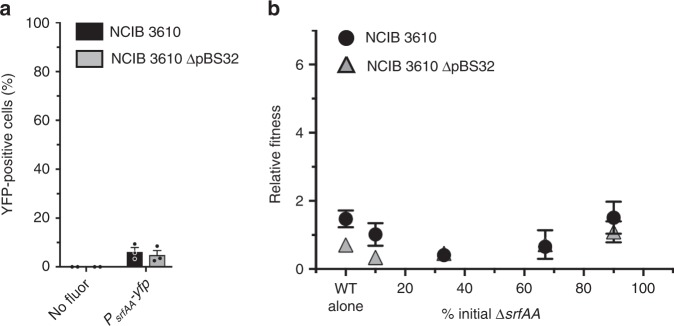
Unicellular growth eliminates differences in public good production and cheating. **a** Percentage of cells expressing the *P*_*srfAA*_*-yfp* transcriptional reporter during logarithmic growth in liquid LB medium. Averages of two (no fluor) or three (*P*_*srfAA*_*-yfp*) biological replicates with SEM. wild type and ∆pBS32 reporters are not statistically different (*P* = 0.641). **b** Relative fitness of ∆*srfAA* in LB co-cultures with wild type in log phase; each point is the mean ± SEM of independent biological replicates (wild-type alone *n* = 4, others *n* = 2). No co-cultures were significantly different from wild-type alone cultures

To more solidly link surfactin production levels to cheater susceptibility, we examined the two phenotypes in a unicellular condition in which cells are not producing public goods: logarithmic growth in liquid LB, a rich complex medium in which having RapP(N236T) can impart a growth advantage^75^. In this context neither NCIB 3610 nor NCIB 3610 ∆pBS32 expressed *srfAA* much above background levels (Fig. 4a, Supplementary Fig. 3B). Concordantly, a ∆*srfAA* mutant had no fitness advantage at any starting frequency in either strain (Fig. 4b). This further supports a causal link between levels of surfactin produced and the ability of a non-producer to cheat, and also shows that cheating is multicellularity-specific.

The differences seen in *srfAA* expression between NCIB 3610 and PS-216 sheds light on their different phenotypes when mixed with ∆*srfAA* cells. Since expression is so low in NCIB 3610, adding non-producers drops it below some critical threshold for efficient swarming and the total yield decreases. Conversely, a PS-216 swarm with added ∆*srfAA* cells mimics NCIB 3610 in that total surfactin production is lowered, which seems to be more efficient since mixed swarms had up to two-fold higher yield (Fig. 1b). Minimizing public good production is thus a better overall use of resources on top of protecting against cheater invasion. Additionally, we think that the protective effect seen from lowering surfactin production is not due to its role as a signaling molecule^80^, as transcription of its downstream target genes *epsA* and *tapA* was not different between strains (Fig. 3b).

### Cheater prevention by cheater induction

**Fig. 5:**
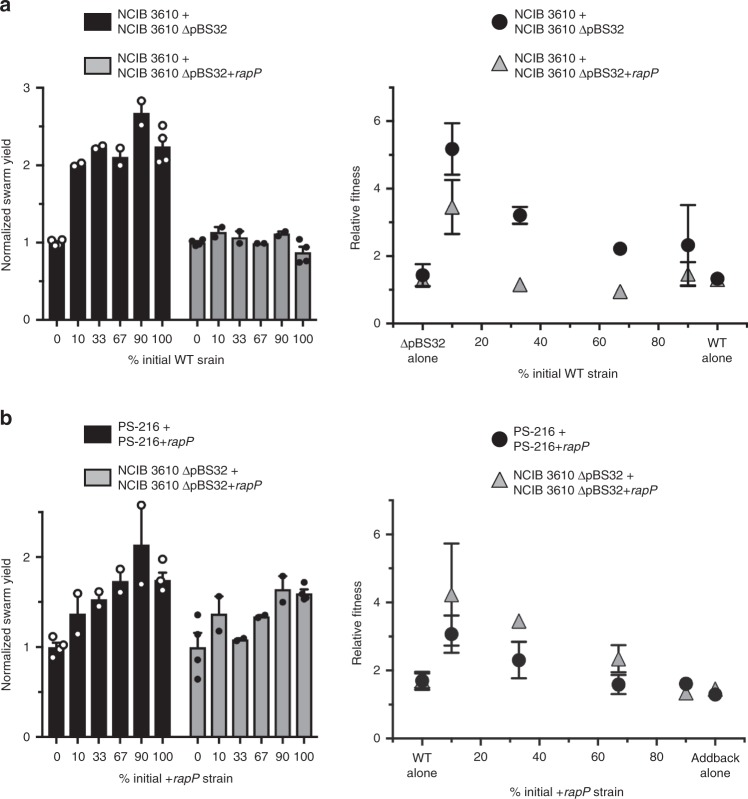
RapP turns host cells into cheaters. **a** Cheating assays in NCIB 3610, except inoculating ∆pBS32 swarms with increasing proportion of wild-type cells instead of ∆*srfAA*. OD_600_ readings of total swarm yield, normalized to the value of 0% wild type. Relative fitness of the wild-type strain when mixed with ∆pBS32 with or without *rapPphrP* added in to the chromosome. The only swarm yields significantly different from 0% wild type were from wild type+∆pBS32 (all *P* < 0.0001). The wild type + ∆pBS32 fitness measurements of 10% and 33% were statistically significant (*P* < 0.0001 and 0.0008), as was the wild type + addback 10% (gray triangle) from both ∆pBS32-alone (*P* < 0.0001) and from wild type + ∆pBS32 10% (black circle, *P* = 0.0042). **b** Cheating assays with PS-216 or NCIB 3610 ∆pBS32 combined with increasing proportion of cells containing *rapPphrP* inserted into the chromosome (*+rapP* strain). All swarm yields were statistically significant except ∆pBS32 33% (*P* = 0.507), as were fitness values of PS-216 10% (*P* = 0.0080) and ∆pBS32 10% and 33% (*P* < 0.0001 and 0.0009). All points in figure are averages of at least two biological replicates, error bars represent SEM.

Because RapP reduces *srfAA* expression so much, most of the cells in an NCIB 3610 swarm are effectively non-producers—phenotypically ∆*srfAA*. We therefore reasoned that NCIB 3610 cells should act as cheaters in the presence of NCIB 3610 ∆pBS32 cells that are paying a high production cost, similar to the growth advantage in liquid LB previously reported^75^. Indeed, the ∆pBS32 strain showed signs of being exploited by wild type in both total swarm yield and negative frequency-dependent fitness disadvantage (Fig. 5a, black bars and circles). This was largely abrogated when wild type was instead mixed with ∆pBS32 + *rapP* (Fig. 5a, gray bars and triangles), indicating the effects are entirely due to the presence of RapP in wild type. The lowest starting percent of wild type did still show some fitness benefit in this last experiment though, which could be due to copy number differences: one *rapPphrP* on the chromosome versus 2 copies of pBS32 per cell^72^.

We next tested whether inserting *rapPphrP* into strains was sufficient to turn them into cheaters. In both the PS-216 and NCIB 3610 ∆pBS32 backgrounds, combining with RapP-containing strains conferred a fitness advantage and increased cell yields, indicative of cheating (Fig. 5b). The phenotypes were not as dramatic as combinations with either a true ∆*srfAA* mutant or the wild-type NCIB 3610, which is likely again due to the lower copy number of chromosomally encoded *rapP* and that 5.05–12.3% of cells still make surfactin.

There are two obvious differences worth pointing out between the experiments in Fig. 5 and the previous assays: (1) here it is the normal wild-type strain that had a fitness advantage, not an engineered mutant and (2) the swarm yields did not go back to zero with higher amounts of cheaters. This is a crucial point, because while the RapP-containing wild-type cells have an advantage and thus could take over a population of no-RapP cells, they are not pure cheaters because they would not collapse the population. NCIB 3610 is thus a nonobligate variation of a facultative cheater, although unlike regulated facultative cheats^45,64,82,83^, its behavior does not change when in the minority versus majority and so does not become cheatable itself.

### Prevalence of *rapP* and N236 mutations

The clear advantage of strains with RapP(N236T) led us to investigate how prevalent this allele is, as we would expect it to spread through a population in a cheater-like way. We started by searching publically available *B. subtilis* genomes for homologs of *rapP* and found 16 hits in 112 unique genomes (14.3%, Table 2), including one on a plasmid that is very similar to pBS32, pLS32 (ref. ^69^). Because genome entries do not always contain plasmid sequences or are not fully assembled, this search could easily have missed many genes. We therefore directly checked for *rapP* by PCR in 83 wild isolates, many of which were isolated on swarm- or biofilm-inducing media^13^, and this time found 27 *rapP* homologs (32.5%, Table 2). This higher incidence could be due to the incompleteness of genome sequences or the source of the strains (many sequenced genomes are from industrially relevant strains), or that some of our strains were selected on multicellularity-inducing media. Regardless, the two methods together uncovered 43 homologs out of 195 strains (22.1%, listed in Supplementary Data 2), indicating that *rapP* is not an uncommon gene.

**Table 2:** Prevalence of *rapP* and N236 mutations in *Bacillus subtilis* strains

			Residue at position 236			
	Strains examined	Have rapP	Asn	Thr	None^a^	Truncation after Asn236
BLAST whole genomes	112	16	15	0	1	1
PCR wild isolates	83	27	24	1	2	3
Total	195	43	39	1	3	4
%		22.1	90.7	2.3	7.0	9.3

^a^Due to either deleted region or upstream frameshift

Looking closer at the identified *rapP* genes, we found that only NCIB 3610 contained the N236T mutation. However, there were seven strains that were either missing residue 236 (resulting from an upstream frameshift or deletion of the region) or had a truncation shortly downstream of 236 (Table 2). These could potentially have the same effect as N236T (PhrP insensitivity), but may also disrupt substrate target binding^84,85^. Moreover, there could be additional residues whose mutation would prevent PhrP binding, as the peptide contacts a number of highly conserved side chains^84,85^, so we cannot rule out other cheater-protective alleles in these strains.

Because Asn236 is highly conserved not just in RapP but in all other Rap proteins^71^, we expanded our search for potential cheater-resistant N236 mutations using a database of 2921 identified *Bacillus* Rap homologs^76^. Searching through the alignment of all full-length Rap proteins, we found mutations at the equivalent of position 236 in 194 Raps from 166 different strains (Table 3, Supplementary Data 2). Most of these occurred outside the *subtilis* group of species: in the 83 closest strains only four of the 881 Raps were mutated. Beyond those immediate species, though, N236 mutations are fairly common or even ubiquitous: of the remaining eight species with more than one representative strain, four have at least one N236 mutation in every single strain, and three more have mutations in at least 49% of strains (Table 3). In the only sequenced *B. clausii* genome, all six distinct Rap homologs have mutations at this position; this species is an outlier however, as only 28 out of the 166 strains have multiple mutant Raps. In total, almost 44% of all sequenced *Bacillus* strains contain a potentially cheater-preventing *rap* allele, and over 57% outside the *subtilis* clade (Table 3). This is likely an underestimate too, since the data only included full-length Rap proteins (excluding potentially interesting truncations), other mutations could produce this effect, and many of the genome accessions searched may not include plasmid sequences, an important source of Rap diversity via horizontal gene transfer (HGT)^76^.

**Table 3:** Bacillus Rap proteins mutated at position 236, by species and amino acid mutation

Species	Thr	Ser	Tyr	Asp	His	Ala	Ile	Total	Orphans (no Phr)	Strains examined	Paralogs^a^	Strains with N236 mutant (%)
*B. subtilis*	2		1	1				4	2	44	1	6.82
*B. mojavensis*								0	0	3	0	0
*B. amyloliquefaciens*								0	0	32	0	0
*B. atropheaus*			3	3				6	3	4	2	100
*B. licheniformis*				9				9	0	13	0	69.2
*B. sonorensis*				1	2			3	2	2	1	100
*B. stratosphericus*				2				2	1	1	1	100
*B. pumilus*		1		13				14	10	11	3	100
*B. safensis*				1				1	1	1	0	100
*B. mycoides*								0	0	3	0	0
*B. cereus*	67	17					1	85	21	147	13	49.0
*B. thuringiensis*	17	6						23	10	25	4	76.0
*B. anthracis*	26							26	1	26	0	100
*B. clausii*	2	1				3		6	6	1	5	100
Total	119	26	4	39	2	3	1	194	62	369	32	43.9

^a^N236 mutant Raps found in same genome as another N236 mutant

When we looked at all 194 of the mutated Rap proteins phylogenetically, they segregated according to the two main *Bacillus* clades, *subtilis* and *cereus*, with *B. clausii* as an outlier group (Supplementary Fig. 5). Even though it is less numerous, the *subtilis* clade is more diverse, representing six Rap clusters to *cereus*’ two, as defined by the original study^76^. Of the 141 *cereus* clade proteins with N236 mutations, all but one are mutated to Thr or Ser (Table 3). This could be reflective of extensive HGT among this group of species, or a lack of diversity among sequenced strains. To this point, all 26 mutant Raps in *B. anthracis* strains have identical amino acid sequences and only one nucleotide difference (Supplementary Data 2). Among *subtilis* clade Raps, the most common mutation at position 236 is Asp, which appears to have been independently mutated at least twice and is found in several species (Table 3, Supplementary Fig. 5). While this residue is overall very well conserved (Asn in 93.4% of Raps, one of only eight residues with that level of conservation), it has been repeatedly and independently mutated to many different amino acids (all of which are potentially one base-pair away from the parent Asn codon).

Another way to achieve constitutive Rap activity could be loss of the associated *phr* gene. These orphan Raps are relatively common, especially in the *subtilis* clade^76^, though due to crosstalk they can sometimes still be inhibited by other Phr peptides^76,84^. If the N236 mutations we have highlighted here abrogate Phr binding like they do in RapP, then we would expect less selection pressure to maintain the downstream *phr* gene. Of the 194 Raps with Asn mutations, 62 do not have an associated *phr* gene (32.0%; Table 3, Supplementary Fig. 5, Supplementary Data 2), which is a significant enrichment over wild-type-236 Raps (712/2727, 26.1%; *P* = 0.0011 by nonparametric two-tailed *t*-test). It could however be the reverse: after a *phr* gene is lost there is relaxed selection on the N236 residue, as there is a higher incidence of Asn mutants among orphan than non-orphan Raps (10.2% versus 6.21%, *P* = 0.0011). Both scenarios result in an over-active repressor, which could target regulators other than Spo0F and ComA. Indeed, some of the mutant Raps are homologous to an allele that did not exhibit activity towards either of these targets in a heterologous system^76^, so while mutation of this residue likely does not have the same phenotype in all organisms, the production of various goods could be minimized with important evolutionary consequences.

## Discussion

In this study we have shown that different *B. subtilis* strains have different responses to the presence of a surfactin-non-producing cheater in swarms, which is due to a single mutation in a plasmid-borne gene that minimizes production of the public good surfactin. Cells with this plasmid gene therefore act similarly to cheaters and could rapidly spread through a population but, importantly, after they take over the cooperative trait is still maintained and in fact more efficient. This strain is thus an optimized cooperator that acts as a nonobligate cheater similar to facultative cheaters^45,64,82,83,86–88^, though this minimal-production approach differs in that the cells do not change strategies upon becoming the majority and so do not become vulnerable to cheating themselves. The strategy is further beneficial because it mitigates cheater mutants that spontaneously arise from within the population, which complements the *B. subtilis* kin discrimination system that protects against newly-encountered populations that might steal public goods^13^. One drawback, though, is that because it is already essentially at the minimum level of surfactin needed to swarm, it has a smaller margin for error and is hurt more by introduced cheaters.

The mechanism uncovered here is reminiscent of previously observed cheater control strategies based on prudent production of cheatable goods. However, those strategies, all described in *P. aeruginosa* and many resulting from experimental evolution rather than naturally occurring mechanisms, are either based on stopping production when it is not needed^16,18^ or being willing to reduce the benefit gained from cooperation by down-regulating production when it could be needed^17,19–21^, trading off maximal cooperativity for cheater protection. The *B. subtilis* system does not make such sacrifices because instead of going below the threshold for maximum cooperative gain, it just avoids overproduction. And since NCIB 3610 swarms just as quickly and makes full use of surfactin’s signaling properties (matrix gene expression is unaffected), it appears that nothing is lost by reducing production. There is a side-effect of less sporulation in this strain (Fig. 3), but it is not known how many spores are optimal and thus whether this could also be avoiding overproduction.

Division of labor is thought to be one of the key advantages of multicellularity because it is more efficient than every individual performing all functions independently^89^. While the flow cytometry profiles of surfactin gene expression in swarms do not suggest true division of labor, the levels in NCIB 3610 are so low that much of the population are essentially non-producers that depend on other cells to produce more surfactin for the entire group, creating a pseudo-division of labor. This was mimicked in the high-production PS-216 strain when ∆*srfAA* cells were added, artificially forcing population heterogeneity and enhancing swarm output, similar to biofilms that were forced into a genetic division of labor^90^ or *Salmonella* virulence heterogeneity during infection^91^. Optimized public good production is thus beneficial not only for efficient allocation of tasks but also as a means of preventing true cheaters from taking over.

Based on our results, the Rap family of regulators seems to be an easy route to achieve minimal production with a single mutation in their Phr binding site—though it means giving up quorum sensing to do so. Given that *subtilis* group and *cereus* group strains average 11 and 6 Raps per genome, respectively^76^, it seems possible for cells to have it both ways: maintain Phr binding in most Raps so regulons can be quorum controlled, but also have one copy mutated to prevent cheater infiltration. We cannot know whether all the mutations we found mitigate cheating because the targets for regulation may differ between species, but the principle of minimal production is translatable to other contexts. Even if the targets are not cheatable goods like surfactin, minimizing the transcription of other key genes could impart interesting behaviors like bet hedging or cell differentiation that may have other meaningful consequences on species’ evolution. Alternatively, these mutations could protect against crosstalk from other Phr peptides, which are often found in multiple copies presumably to manipulate other cells’ altruistic behaviors^64^. Lastly, we note that because minimized expression via Rap proteins economizes production costs, it might not be purely selected for its effect on sociality.

The virtual absence of N236 mutations in *B. subtilis* strains is surprising (Table 3), and could be a result of other cheater protection mechanisms at work, such as the diversification of surfactants in the *subtilis* clade^92,93^ (though this diversity was shown to affect biofilms more than swarms^94^). It may also suggest that NCIB 3610, the supposedly wild strain commonly used in lab studies, is likely at least partially domesticated from its original Marburg ancestor^75^ based on a recent genomic analysis that found an amount of gene loss similar to that of lab strains^95^. Nevertheless, our results have still uncovered a molecular mechanism by which cheating can be prevented, even if it did not originate in the wild (although surely not all of the 194 Asn mutants in Table 3 are the result of domestication). If the selection for the RapP-N236T mutation in NCIB 3610 was artificial, it could still have been due to its cheater control benefits, as laboratory growth conditions involve larger populations than natural settings and thus more opportunity for cheaters to arise and more pressure on public goods producers. We know that the plasmid carrying this gene was lost upon further domestication in the derived lab strain 168, along with other public good genes and regulators necessary for biofilm formation^68^. This explains why previous papers were able to demonstrate cheating in a lab strain^45,64^ while we do not see it in the parent NCIB 3610, underscoring the importance of using multiple wild strains, especially when studying social traits.

## Methods

### Growth conditions

Strains used in this study are listed in Supplementary Table 1. To initiate swarming, saturated LB cultures were diluted to OD_600_ = 0.5 and 2 µl spotted in the center of a low-agar B medium plate (50 mM Tris-HCl pH 7.5, 0.6 mM KH_2_PO_4_, 27 mM KCl, 15 mM (NH_4_)_2_SO_4_, 7 mM Na citrate, 2 mM CaCl_2_, 1 μM FeSO_4_, 8 mM MgSO_4_, 10 μM MnSO_4_, 4.5 mM Na glutamate, 0.2% glucose, 0.7% agar) that had been dried in a laminar flow hood for 30 min; plates were then incubated in a sealed container at 30 ˚C overnight (16 h). For the initial swarm expansion rate measurements in Supplementary Fig. 1B, overnight LB cultures were first spun and washed to remove surfactin in the liquid medium, then 10× more cells than normal (OD_600_ = 5) were spotted on B medium at 37˚C in order to initiate spreading sooner. For unicellular conditions in Fig. 4, overnight cultures were diluted to OD_600_ = 0.001 in 15 ml of LB and grown to OD_600_ = 0.5–1.0, approximating the increase in cell number that occurs on swarm plates while still staying in the logarithmic phase.

### Cheating assays

Strains constitutively expressing different fluorescent protein combinations (RFP alone or RFP plus GFP) were combined in varying initial proportions and spotted on swarm-inducing plates as above. After overnight incubation, the entire swarm was scraped off the agar surface, suspended in 1 ml phosphate-buffered saline (PBS; 1.7 mM KH_2_PO_4_, 5 mM Na_2_HPO_4_, 150 mM NaCl), vortexed, passed through a 23-gauge needle 10× to break up clumps, sonicated 15× on ice at 20% amplitude, washed once, and resuspended in 1 ml PBS. Swarm yield was determined by OD_600_ readings of this suspension on a spectrophotometer, and normalized to the values of wild type alone to reduce day-to-day variation. Final proportions of each strain were measured by flow cytometry, and relative fitness was determined by the following formula: [*f*/(1−*f*)]/[*i*−(1−*i*)], where *f* is the final proportion and *i* the initial proportion. Spatial distributions of strains constitutively expressing RFP or YFP were visualized on a fluorescent stereoscope and analyzed by ImageJ software. All experiments were repeated with the fluorescent combinations swapped to control for any effect of the markers on fitness, and wild type + wild-type controls (0% mutant) at all tested ratios were included in every experiment.

### Gene expression analysis

Swarms were grown in triplicate as above, except strains were diluted 100× to OD_600_ = 0.005 before spotting on swarming plates in order to capture early-development dynamics, scraped into 400 µl RNAlater solution, vortexed, passed through a needle 10×, incubated for 15 min at room temperature, then sonicated 25× with 1 s pulses at 30% amplitude. After washing once in phosphate-buffered saline, cells were incubated in 200 µl of 15 mg/ml lysozyme in 37.5 mM Tris (pH 7.5) plus 2 mM EDTA for 2 h at room temperature. After adding 400 µl RLT buffer (Qiagen) containing 1:100 β-mercaptoethanol, lysates were diluted ten-fold in RLT for hybridization with probeset. Isolation and quantification of RNA by NanoString nCounter SPRINT was done according to the manufacturer’s instructions^77^. Abundances of each mRNA were normalized to housekeeping gene counts, averaged among the three biological replicates, and any strain ratios greater than two-fold and *t*-test >0.05 were considered significant. See Supplementary Data 1 for full results.

### Transcriptional reporter assays

Strains containing constructs with different promoters in front of the *yfp* gene were spotted on swarming plates at OD_600_ = 0.005, except *P*_*sspB*_*-yfp* strains were spotted at the normal concentration since sporulation occurs later. Swarms were grown overnight at 30 ˚C, scraped into 0.5 ml PBS, vortexed, passed through a needle 10×, fixed in 4% paraformaldehyde at room temperature for 7 min to maintain the expression level in the swarm, washed in 0.5 ml PBS, resuspended in 0.5 ml GTE (50 mM glucose, 20 mM Tris pH 8.0, 10 mM EDTA), and sonicated 15× on ice at 20% amp. Cells were diluted to ~OD_600_ = 0.3 and run on an LSR-II flow cytometer using FACS Diva software. Tight forward- and side-scatter gates were used to filter out clumps of cells, and controls of single-fluorophore (cheating assays) or no-fluorophore (reporter assays) were used to identify the different fluorescent populations. Histograms in Supplementary Fig. 3A–C were made in FCS Express 6 software, and the % YFP-positive cells shown in Fig. 3 are the percentage of events that remained after subtracting out the no-fluorescence control histograms. Median YFP levels in Supplementary Fig. 3D were calculated before histogram subtraction and divided by the median value of the no-fluorescence control done on the same day.

### Surfactant quantification

Fully-developed swarms from three replicates were scraped off of plates and resuspended in 200 µl PBS, OD_600_ readings were taken, then cells were pelleted, and supernatants harvested into new tubes. Supernatants were then serially diluted and 5 µl spotted onto parafilm, allowed to settle for 10 min, photographed, and diameters of spots were correlated to dilutions of purchased surfactin (Sigma) to estimate the total concentration of surfactants in the swarms. To normalize by the number of cells in each swarm, OD_600_ readings were converted to CFUs using Supplementary Fig. 1A. Total surfactant in the swarms was statistically different between NCIB 3610 and PS-216 (*P* = 0.0102) and NCIB 3610 ∆pBS32 (*P* = 9.76 × 10^−4^), and between PS-216 and NCIB 3610 ∆pBS32 (*P* = 0.0178); and surfactant levels normalized to cell counts were significantly different between NCIB 3610 and PS-216 (*P* = 0.0174) and NCIB 3610 ∆pBS32 (*P* = 0.00107), but not between PS-216 and NCIB 3610 ∆pBS32 (*P* = 0.0534) by two-tailed *t*-tests.

### Statistical analyses

In cheating assays, two-way analyses of variance were performed on both the normalized OD_600_ and relative fitness values, comparing each ratio to wild-type alone within each strain using uncorrected Fisher’s LSD test. For comparisons between strains (absolute OD_600_, all gene expression assays, and surfactant quantification), two-tailed *t*-tests were used without assuming consistent standard deviations. All statistical tests were done in Prism v7.0 software.

### rapP PCR and BLAST

Primers specific to the N236 region of *rapP* (Fwd CCATGAATTATGCTCAGCGAGC, Rev CTTCCTGGTTGTTGTGCCGG; 434 bp) were reacted with genomic DNA from our collection of wild strains to detect and sequence potential homologs, using DNA from NCIB 3610 and NCIB 3610 ∆pBS32 as positive and negative controls in each set of reactions. Because only the interior of the ORF was amplified, we cannot know if the homologs all contain the same *phrP* immediately downstream. For BLASTn searches, full-length *rapP* gene (GenBank: CP020103.1, nucleotide positions 29607–30770) was used to query *Bacillus subtilis* (taxid: 1423) nr/nt and WGS databases, requiring >90% identity over >90% of the gene to be considered a homolog. Lab strains, contaminated genome sequences, and duplicate strains between the two approaches were excluded from final numbers. Homologs were confirmed by clustering in a phylogenetic tree with *rapP* and not the nearest homolog *rapI* (Supplementary Fig. 6). All strains examined by PCR and all strains with homologs found by BLAST are listed in Supplementary Data 2.

### N236 mutant analysis

The alignment of all Raps taken from ref. ^76^ was manually scanned for substitutions at the position that aligned to 236 of RapP. These non-asparagine mutant sequences were then isolated and re-aligned for phylogenetic analysis in Mega v6.06. The percentage of strains with a mutant Rap was determined by first subtracting out the number of paralogs from the total number of mutated Raps in each species, then dividing by the number of strains examined. Unnamed species and species with a single representative are not listed in Table 3 for simplicity but were included in the totals. Supplementary Data 2 contains every species, strain, Rap ID number, 236 residue, and amino acid sequence of the identified mutant Raps and the Phr immediately downstream (if any).

## Electronic supplementary material

Supplementary Information

Data Set 1

Data Set 2

Description of Additional Supplementary Items

## Data Availability

All datasets generated and analyzed in this study are contained in the article and supplementary information files.

## References

[1] Strassmann JE, Queller DC (2011). Evolution of cooperation and control of cheating in a social microbe. Proc. Natl. Acad. Sci. USA.

[2] Ozkaya, O., Xavier, K. B., Dionisio, F. & Balbontin, R. Maintenance of microbial cooperation mediated by public goods in single and multiple traits scenarios. *J. Bacteriol.*10.1128/JB.00297-17 (2017). 10.1128/JB.00297-17 PMC5648865 28847922

[3] Amherd M, Velicer GJ, Rendueles O, Holman L (2018). Spontaneous nongenetic variation of group size creates cheater-free groups of social microbes. Behav. Ecol..

[4] Bastiaans E, Debets AJ, Aanen DK (2016). Experimental evolution reveals that high relatedness protects multicellular cooperation from cheaters. Nat. Commun..

[5] Brockhurst MA (2007). Population bottlenecks promote cooperation in bacterial biofilms. PLoS ONE.

[6] Diggle SP, Griffin AS, Campbell GS, West SA (2007). Cooperation and conflict in quorum-sensing bacterial populations. Nature.

[7] Gilbert OM, Foster KR, Mehdiabadi NJ, Strassmann JE, Queller DC (2007). High relatedness maintains multicellular cooperation in a social amoeba by controlling cheater mutants. Proc. Natl. Acad. Sci. USA.

[8] Ho HI, Hirose S, Kuspa A, Shaulsky G (2013). Kin recognition protects cooperators against cheaters. Curr. Biol..

[9] Ho HI, Shaulsky G (2015). Temporal regulation of kin recognition maintains recognition-cue diversity and suppresses cheating. Nat. Commun..

[10] Inglis RF, Ryu E, Asikhia O, Strassmann JE, Queller DC (2017). Does high relatedness promote cheater-free multicellularity in synthetic lifecycles?. J. Evol. Biol..

[11] Koschwanez JH, Foster KR, Murray AW (2011). Sucrose utilization in budding yeast as a model for the origin of undifferentiated multicellularity. PLoS Biol..

[12] Kuzdzal-Fick JJ, Fox SA, Strassmann JE, Queller DC (2011). High relatedness is necessary and sufficient to maintain multicellularity in *Dictyostelium*. Science.

[13] Lyons, N. A. & Kolter, R. Bacillus subtilis protects public goods by extending kin discrimination to closely related species. *mBio* **8**, 10.1128/mBio.00723-17 (2017). 10.1128/mBio.00723-17 PMC5573675 28679746

[14] Queller DC, Ponte E, Bozzaro S, Strassmann JE (2003). Single-gene greenbeard effects in the social amoeba *Dictyostelium discoideum*. Science.

[15] Shapiro-Ilan D, Raymond B (2016). Limiting opportunities for cheating stabilizes virulence in insect parasitic nematodes. Evol. Appl..

[16] Ghoul M (2016). Pyoverdin cheats fail to invade bacterial populations in stationary phase. J. Evol. Biol..

[17] Gupta R, Schuster M (2013). Negative regulation of bacterial quorum sensing tunes public goods cooperation. ISME J..

[18] Kummerli R, Brown SP (2010). Molecular and regulatory properties of a public good shape the evolution of cooperation. Proc. Natl. Acad. Sci. USA.

[19] Kummerli R (2015). Co-evolutionary dynamics between public good producers and cheats in the bacterium Pseudomonas aeruginosa. J. Evol. Biol..

[20] O’Brien, S., Lujan, A. M., Paterson, S., Cant, M. A. & Buckling, A. Adaptation to public goods cheats in Pseudomonas aeruginosa. *Proc. Biol. Sci.* **284**, 10.1098/rspb.2017.1089 (2017). 10.1098/rspb.2017.1089 PMC5543229 28747481

[21] Xavier JB, Kim W, Foster KR (2011). A molecular mechanism that stabilizes cooperative secretions in Pseudomonas aeruginosa. Mol. Microbiol..

[22] Drescher K, Nadell CD, Stone HA, Wingreen NS, Bassler BL (2014). Solutions to the public goods dilemma in bacterial biofilms. Curr. Biol..

[23] Julou T (2013). Cell-cell contacts confine public goods diffusion inside Pseudomonas aeruginosa clonal microcolonies. Proc. Natl. Acad. Sci. USA.

[24] Kummerli R, Griffin AS, West SA, Buckling A, Harrison F (2009). Viscous medium promotes cooperation in the pathogenic bacterium Pseudomonas aeruginosa. Proc. Biol. Sci..

[25] Mund, A., Diggle, S. P. & Harrison, F. The fitness of Pseudomonas aeruginosa quorum sensing signal cheats is influenced by the diffusivity of the environment. *MBio* **8**, 10.1128/mBio.00353-17 (2017). 10.1128/mBio.00353-17 PMC5414003 28465424

[26] Scholz RL, Greenberg EP (2015). Sociality in Escherichia coli: enterochelin is a private good at low cell density and can be shared at high cell density. J. Bacteriol..

[27] Bruce JB, Cooper GA, Chabas H, West SA, Griffin AS (2017). Cheating and resistance to cheating in natural populations of the bacterium Pseudomonas fluorescens. Evolution.

[28] Dandekar AA, Chugani S, Greenberg EP (2012). Bacterial quorum sensing and metabolic incentives to cooperate. Science.

[29] Foster KR, Shaulsky G, Strassmann JE, Queller DC, Thompson CR (2004). Pleiotropy as a mechanism to stabilize cooperation. Nature.

[30] Friman VP, Diggle SP, Buckling A (2013). Protist predation can favour cooperation within bacterial species. Biol. Lett..

[31] Garcia-Contreras R (2015). Quorum sensing enhancement of the stress response promotes resistance to quorum quenching and prevents social cheating. ISME J..

[32] Goo, E., Kang, Y., Lim, J. Y., Ham, H. & Hwang, I. Lethal consequences of overcoming metabolic restrictions imposed on a cooperative bacterial population. *MBio* **8**, https://doi.org/10.1128/mBio.00042-17 (2017). 10.1128/mBio.00042-17 PMC5347341 28246357

[33] Hawver, L. A., Giulietti, J. M., Baleja, J. D. & Ng, W. L. Quorum sensing coordinates cooperative expression of pyruvate metabolism genes to maintain a sustainable environment for population stability. *MBio* **7**, 10.1128/mBio.01863-16 (2016). 10.1128/mBio.01863-16 PMC5142617 27923919

[34] Jousset A, Eisenhauer N, Materne E, Scheu S (2013). Evolutionary history predicts the stability of cooperation in microbial communities. Nat. Commun..

[35] Majerczyk, C., Schneider, E. & Greenberg, E. P. Quorum sensing control of Type VI secretion factors restricts the proliferation of quorum-sensing mutants. *Elife* **5**, 10.7554/eLife.14712 (2016). 10.7554/eLife.14712 PMC4868534 27183270

[36] Manhes P, Velicer GJ (2011). Experimental evolution of selfish policing in social bacteria. Proc. Natl. Acad. Sci. USA.

[37] Mumford R, Friman VP (2017). Bacterial competition and quorum-sensing signalling shape the eco-evolutionary outcomes of model in vitro phage therapy. Evol. Appl..

[38] Saucedo-Mora MA (2017). Selection of functional quorum sensing systems by lysogenic bacteriophages in Pseudomonas aeruginosa. Front. Microbiol..

[39] Smalley NE, An D, Parsek MR, Chandler JR, Dandekar AA (2015). Quorum sensing protects Pseudomonas aeruginosa against cheating by other species in a laboratory coculture model. J. Bacteriol..

[40] Wang M, Schaefer AL, Dandekar AA, Greenberg EP (2015). Quorum sensing and policing of Pseudomonas aeruginosa social cheaters. Proc. Natl. Acad. Sci. USA.

[41] Wilder CN, Diggle SP, Schuster M (2011). Cooperation and cheating in Pseudomonas aeruginosa: the roles of the las, rhl and pqs quorum-sensing systems. ISME J..

[42] Wolf JB (2015). Fitness trade-offs result in the illusion of social success. Curr. Biol..

[43] Butaite E, Baumgartner M, Wyder S, Kummerli R (2017). Siderophore cheating and cheating resistance shape competition for iron in soil and freshwater Pseudomonas communities. Nat. Commun..

[44] Greig D, Travisano M (2004). The prisoner’s dilemma and polymorphism in yeast SUC genes. Proc. Biol. Sci..

[45] Pollak S (2016). Facultative cheating supports the coexistence of diverse quorum-sensing alleles. Proc. Natl. Acad. Sci. USA.

[46] Stilwell, P., Lowe, C. & Buckling, A. The effect of cheats on siderophore diversity in Pseudomonas aeruginosa. *J. Evol. Biol.*, 10.1111/jeb.13307 (2018). 10.1111/jeb.13307 PMC6175192 29904987

[47] Hol FJ (2013). Spatial structure facilitates cooperation in a social dilemma: empirical evidence from a bacterial community. PLoS ONE.

[48] Jack CN (2015). Migration in the social stage of Dictyostelium discoideum amoebae impacts competition. PeerJ.

[49] Lujan AM, Gomez P, Buckling A (2015). Siderophore cooperation of the bacterium Pseudomonas fluorescens in soil. Biol. Lett..

[50] MacLean RC, Gudelj I (2006). Resource competition and social conflict in experimental populations of yeast. Nature.

[51] Momeni B, Waite AJ, Shou W (2013). Spatial self-organization favors heterotypic cooperation over cheating. eLife.

[52] Pande S (2016). Privatization of cooperative benefits stabilizes mutualistic cross-feeding interactions in spatially structured environments. ISME J..

[53] Ross-Gillespie A, Gardner A, Buckling A, West SA, Griffin AS (2009). Density dependence and cooperation: theory and a test with bacteria. Evolution.

[54] Schluter J, Nadell CD, Bassler BL, Foster KR (2015). Adhesion as a weapon in microbial competition. ISME J..

[55] Smukalla S (2008). *FLO1* is a variable green beard gene that drives biofilm-like cooperation in budding yeast. Cell.

[56] Van Dyken JD, Muller MJ, Mack KM, Desai MM (2013). Spatial population expansion promotes the evolution of cooperation in an experimental Prisoner’s Dilemma. Curr. Biol..

[57] van Gestel J, Weissing FJ, Kuipers OP, Kovacs AT (2014). Density of founder cells affects spatial pattern formation and cooperation in Bacillus subtilis biofilms. ISME J..

[58] Yan J, Nadell CD, Stone HA, Wingreen NS, Bassler BL (2017). Extracellular-matrix-mediated osmotic pressure drives Vibrio cholerae biofilm expansion and cheater exclusion. Nat. Commun..

[59] Kearns DB, Chu F, Rudner R, Losick R (2004). Genes governing swarming in *Bacillus subtilis* and evidence for a phase variation mechanism controlling surface motility. Mol. Microbiol..

[60] Stefanic P, Kraigher B, Lyons NA, Kolter R, Mandic-Mulec I (2015). Kin discrimination between sympatric *Bacillus subtilis* isolates. Proc. Natl. Acad. Sci. USA.

[61] Lyons NA, Kraigher B, Stefanic P, Mandic-Mulec I, Kolter R (2016). A combinatorial kin discrimination system in Bacillus subtilis. Curr. Biol..

[62] Dragos, A. et al. Evolution of exploitative interactions during diversification in Bacillus subtilis biofilms. *FEMS Microbiol. Ecol*. **93**, 10.1093/femsec/fix155 (2017). 10.1093/femsec/fix155 29126191

[63] Martin M (2017). De novo evolved interference competition promotes the spread of biofilm defectors. Nat. Commun..

[64] Even-Tov E (2016). Social evolution selects for redundancy in bacterial quorum sensing. PLoS Biol..

[65] Stefanic P, Mandic-Mulec I (2009). Social interactions and distribution of *Bacillus subtilis* pherotypes at microscale. J. Bacteriol..

[66] Carcamo-Oyarce G, Lumjiaktase P, Kummerli R, Eberl L (2015). Quorum sensing triggers the stochastic escape of individual cells from Pseudomonas putida biofilms. Nat. Commun..

[67] Durrett, R. et al. Genome sequence of the *Bacillus subtilis* biofilm-forming transformable strain PS216. *Genome Announc.* **1**, 10.1128/genomeA.00288-13 (2013). 10.1128/genomeA.00288-13 PMC3707585 23788536

[68] McLoon AL, Guttenplan SB, Kearns DB, Kolter R, Losick R (2011). Tracing the domestication of a biofilm-forming bacterium. J. Bacteriol..

[69] Konkol MA, Blair KM, Kearns DB (2013). Plasmid-encoded ComI inhibits competence in the ancestral 3610 strain of Bacillus subtilis. J. Bacteriol..

[70] Parashar V, Konkol MA, Kearns DB, Neiditch MB (2013). A plasmid-encoded phosphatase regulates Bacillus subtilis biofilm architecture, sporulation, and genetic competence. J. Bacteriol..

[71] Omer Bendori S, Pollak S, Hizi D, Eldar A (2015). The RapP-PhrP quorum-sensing system of Bacillus subtilis strain NCIB3610 affects biofilm formation through multiple targets, due to an atypical signal-insensitive allele of RapP. J. Bacteriol..

[72] Myagmarjav BE, Konkol MA, Ramsey J, Mukhopadhyay S, Kearns DB (2016). ZpdN, a plasmid-encoded sigma factor homolog, induces pBS32-dependent cell death in Bacillus subtilis. J. Bacteriol..

[73] Pottathil M, Lazazzera BA (2003). The extracellular Phr peptide-Rap phosphatase signaling circuit of Bacillus subtilis. Front. Biosci..

[74] Lazazzera BA, Solomon JM, Grossman AD (1997). An exported peptide functions intracellularly to contribute to cell density signaling in B. subtilis. Cell.

[75] Pollak S, Omer Bendori S, Eldar A (2015). A complex path for domestication of B. subtilis sociality. Curr. Genet..

[76] Even-Tov E, Omer Bendori S, Pollak S, Eldar A (2016). Transient duplication-dependent divergence and horizontal transfer underlie the evolutionary dynamics of bacterial cell-cell signaling. PLoS Biol..

[77] Geiss GK (2008). Direct multiplexed measurement of gene expression with color-coded probe pairs. Nat. Biotechnol..

[78] Molle V (2003). The Spo0A regulon of Bacillus subtilis. Mol. Microbiol..

[79] Comella N, Grossman AD (2005). Conservation of genes and processes controlled by the quorum response in bacteria: characterization of genes controlled by the quorum-sensing transcription factor ComA in Bacillus subtilis. Mol. Microbiol..

[80] Lopez D, Vlamakis H, Losick R, Kolter R (2009). Paracrine signaling in a bacterium. Genes Dev..

[81] van Gestel J, Vlamakis H, Kolter R (2015). From cell differentiation to cell collectives: Bacillus subtilis uses division of labor to migrate. PLoS Biol..

[82] Santorelli LA (2008). Facultative cheater mutants reveal the genetic complexity of cooperation in social amoebae. Nature.

[83] Madsen JS (2015). Facultative control of matrix production optimizes competitive fitness in Pseudomonas aeruginosa PA14 biofilm models. Appl. Environ. Microbiol..

[84] Parashar V, Jeffrey PD, Neiditch MB (2013). Conformational change-induced repeat domain expansion regulates Rap phosphatase quorum-sensing signal receptors. PLoS Biol..

[85] Gallego del Sol F, Marina A (2013). Structural basis of Rap phosphatase inhibition by Phr peptides. PLoS Biol..

[86] Fiegna F, Yu YT, Kadam SV, Velicer GJ (2006). Evolution of an obligate social cheater to a superior cooperator. Nature.

[87] Gore J, Youk H, van Oudenaarden A (2009). Snowdrift game dynamics and facultative cheating in yeast. Nature.

[88] Healey D, Axelrod K, Gore J (2016). Negative frequency-dependent interactions can underlie phenotypic heterogeneity in a clonal microbial population. Mol. Syst. Biol..

[89] van Gestel J, Vlamakis H, Kolter R (2015). Division of labor in biofilms: the ecology of cell differentiation. Microbiol Spectr..

[90] Dragos, A. et al. Division of labor during biofilm matrix production. *Curr. Biol.*, 10.1016/j.cub.2018.04.046 (2018). 10.1016/j.cub.2018.04.046 PMC6331042 29887307

[91] Diard M (2013). Stabilization of cooperative virulence by the expression of an avirulent phenotype. Nature.

[92] Habe H, Taira T, Imura T (2017). Screening of a Bacillus subtilis strain producing multiple types of cyclic lipopeptides and evaluation of their surface-tension-lowering aActivities. J. Oleo Sci..

[93] Roongsawang N, Washio K, Morikawa M (2010). Diversity of nonribosomal peptide synthetases involved in the biosynthesis of lipopeptide biosurfactants. Int. J. Mol. Sci..

[94] Aleti G (2016). Surfactin variants mediate species-specific biofilm formation and root colonization in Bacillus. Environ. Microbiol..

[95] Brito PH (2018). Genetic competence drives genome diversity in Bacillus subtilis. Genome Biol. Evol..

