# Supplementary material for: A single mutation in rapP induces cheating to prevent cheating in Bacillus subtilis by minimizing public good production
Source: Commun Biol. 2018 Sep 4;1:133. doi: 10.1038/s42003-018-0136-1 (PMC6123732; doi:10.1038/s42003-018-0136-1)
Supplement: Supplementary file 1 — Supplementary Information [file 42003_2018_136_MOESM1_ESM.pdf]

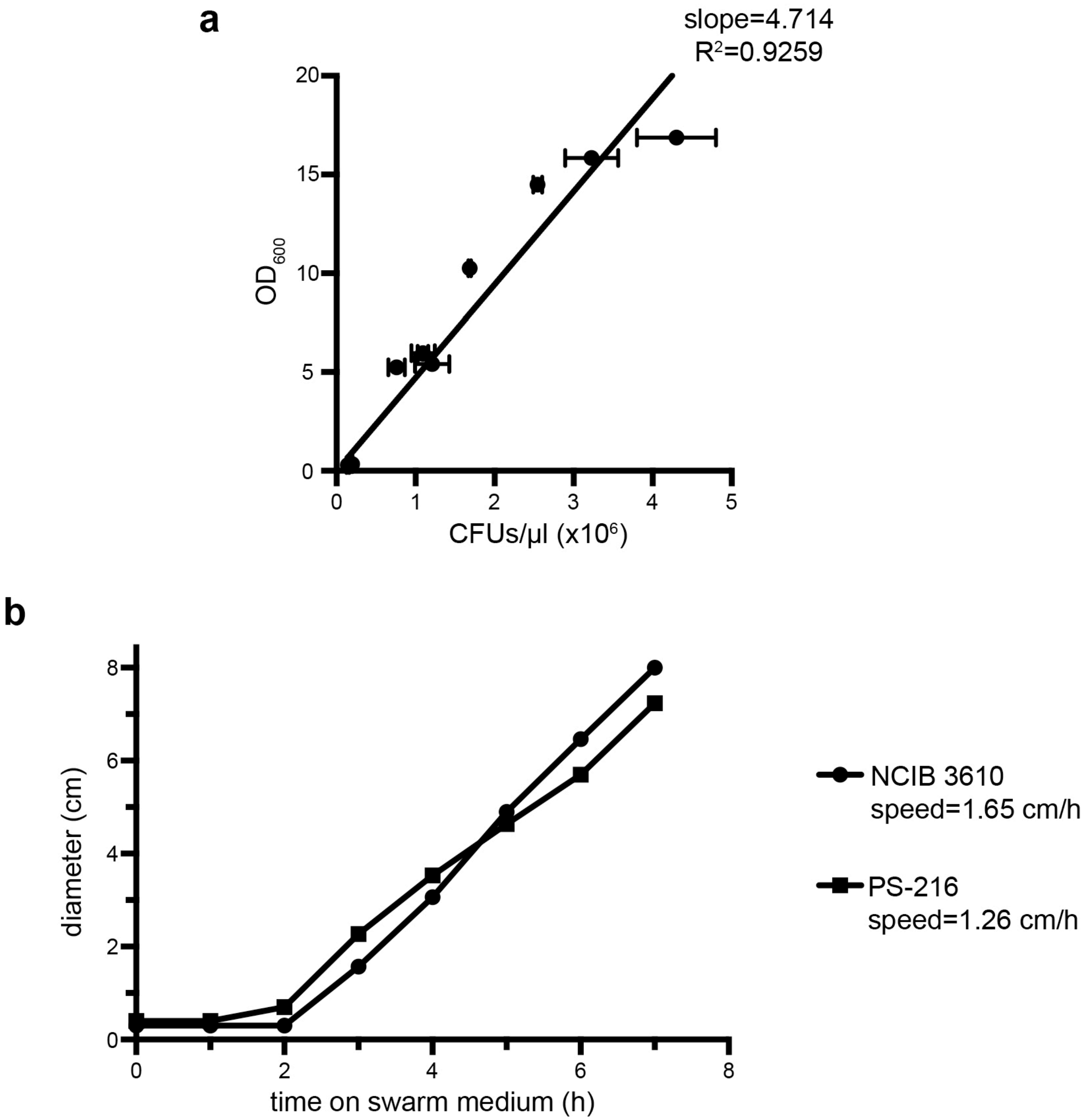

Supplementary Figure 1, related to Figure 1. Justification of OD_{600} measurements as proxy for fitness.

A) Correlation between OD_{600} readings and colony-forming units (CFUs) per µl across a variety of swarm sizes. The slope of the resulting trendline (forced to intercept the origin) as well as the goodness of fit are indicated above the graph.

B) Initial swarm expansion of two strains, with their speeds calculated from the slope of the lines after swarming initiated. Speeds are significantly different by F-test ($P<0.0001$, $F=95.83$). Averages of three replicate swarms; error bars are the SEM but smaller than the symbol size in most instances.

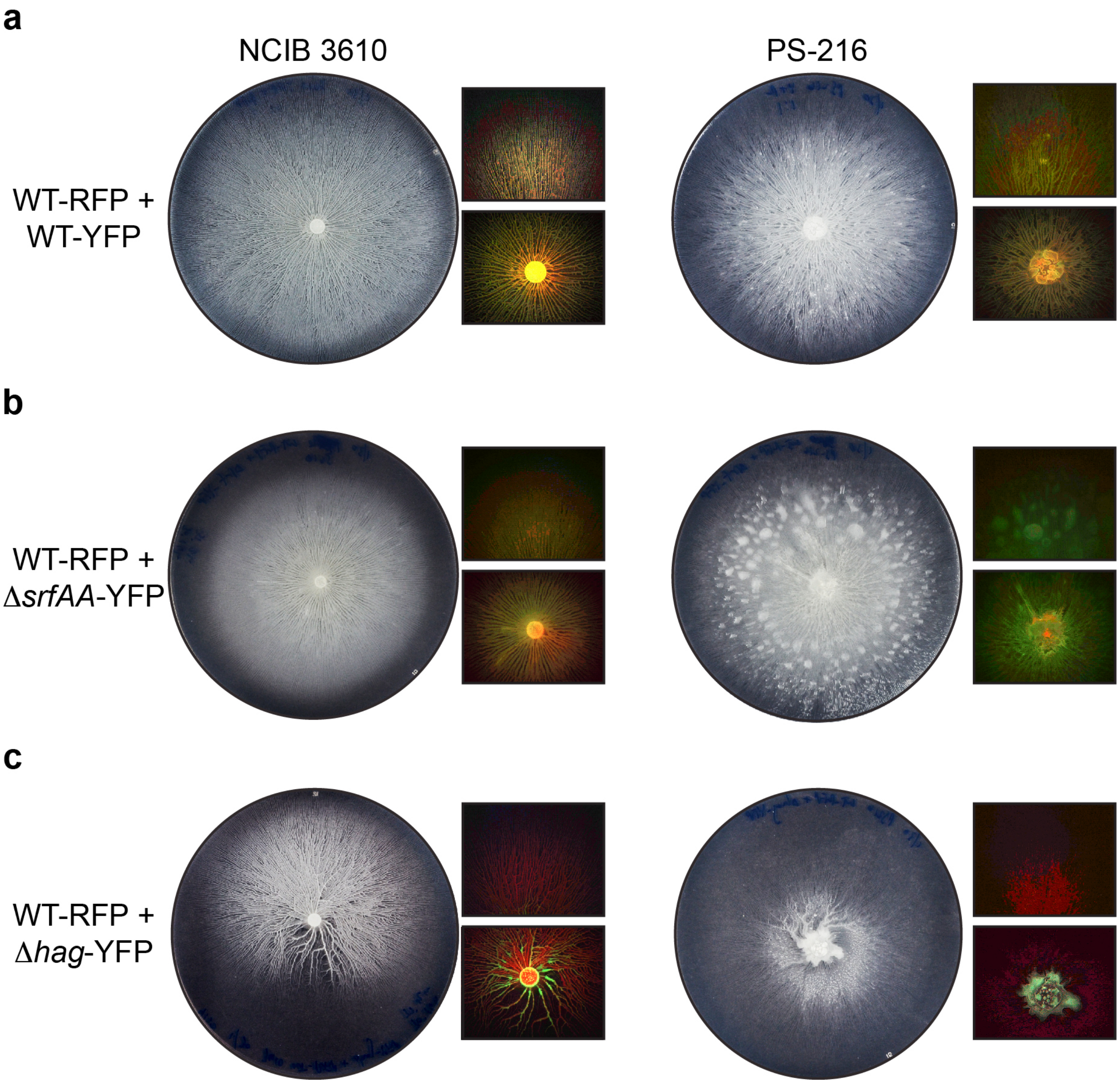

Supplementary Figure 2, related to Figure 1. Spatial distribution of WT and mutant cells within swarms.

WT cells constitutively producing RFP were combined in 1:1 ratios with YFP-producing cells (false-colored green) of either WT (A), Δ*srfAA* (B), or Δ*hag* (C), in both NCIB 3610 and PS-216 strain backgrounds. Magnified areas of the center and top of each plate are shown with merged RFP and YFP fluorescent image captures. Similar results were obtained when fluorescent proteins were swapped (WT-YFP + mutant-RFP).

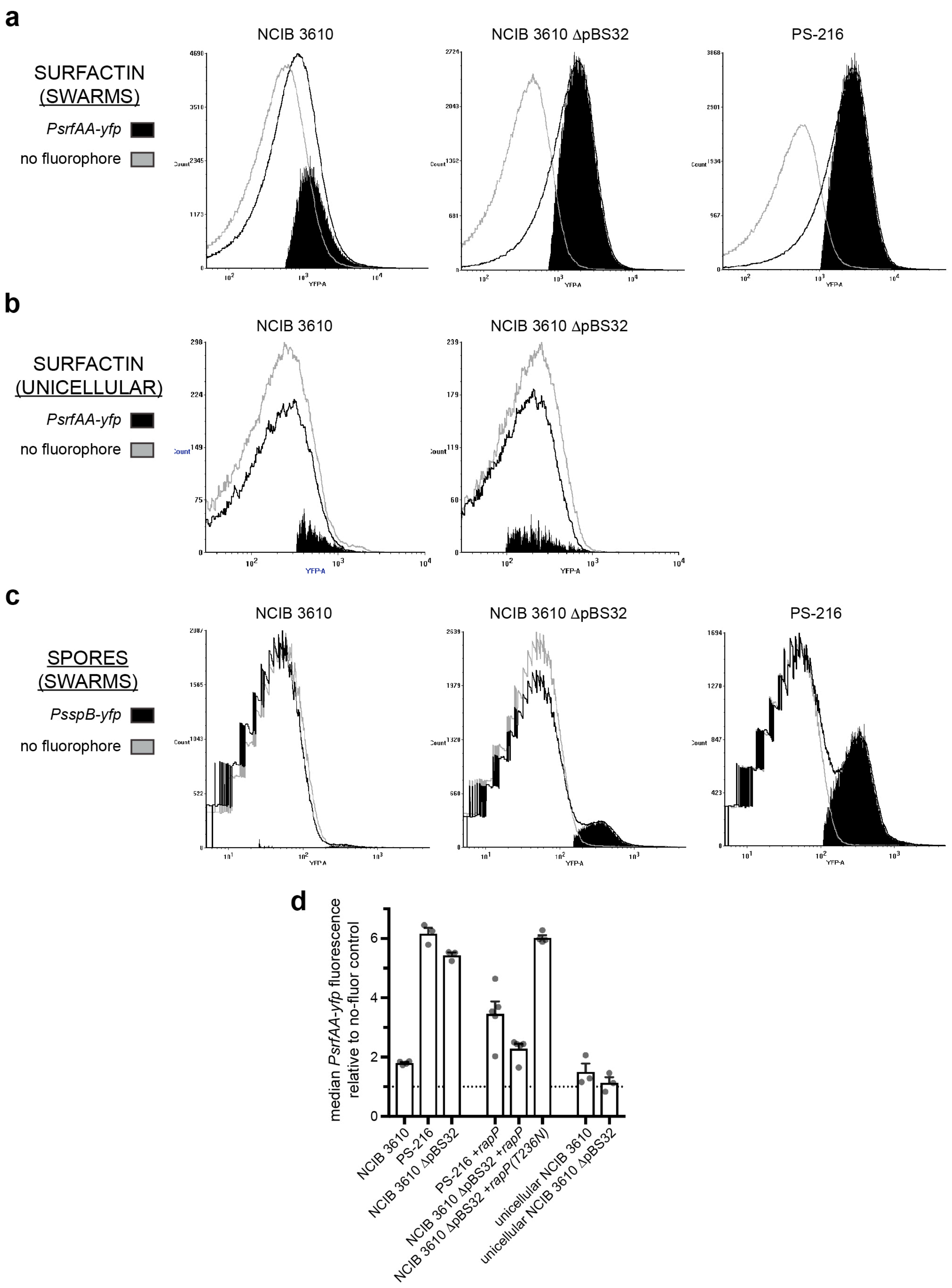
a
SURFACTIN
(SWARMS)
PsrfAA-yfp
no fluorophore
NCIB 3610
NCIB 3610 ΔpBS32
PS-216
b
SURFACTIN
(UNICELLULAR)
PsrfAA-yfp
no fluorophore
NCIB 3610
NCIB 3610 ΔpBS32
c
SPORES
(SWARMS)
PsspB-yfp
no fluorophore
NCIB 3610
NCIB 3610 ΔpBS32
PS-216
d
median PsrfAA-yfp fluorescence relative to no-fluor control
NCIB 3610
PS-216
NCIB 3610 ΔpBS32
PS-216 +rapP
NCIB 3610 ΔpBS32 +rapP
NCIB 3610 ΔpBS32 +rapP(T236N)
unicellular NCIB 3610
unicellular NCIB 3610 ΔpBS32

Supplementary Figure 3, related to Figures 3 and 4. Flow cytometry profiles of transcriptional reporters.

A–C) Representative histograms of YFP fluorescence detected from cells expressing P_{srfAA}-*yfp* in swarms (A) or liquid LB (B), or P_{sspB}-*yfp* in swarms (C) in the indicated strain backgrounds. Gray lines are control strains with no fluorescent construct, black lines are the reporter strains, and the filled black areas are what remains of the reporter profile after subtracting out the no-fluorophore histogram to leave only the definitively YFP-positive events.

D) The P_{srfAA}-*yfp* flow cytometry data from Figure 3 presented as the median YFP fluorescence levels relative to the median of the no-fluorescence controls (indicated by the dashed line at 1) without any histogram subtraction. Averages of three biological replicates, error bars represent SEM.

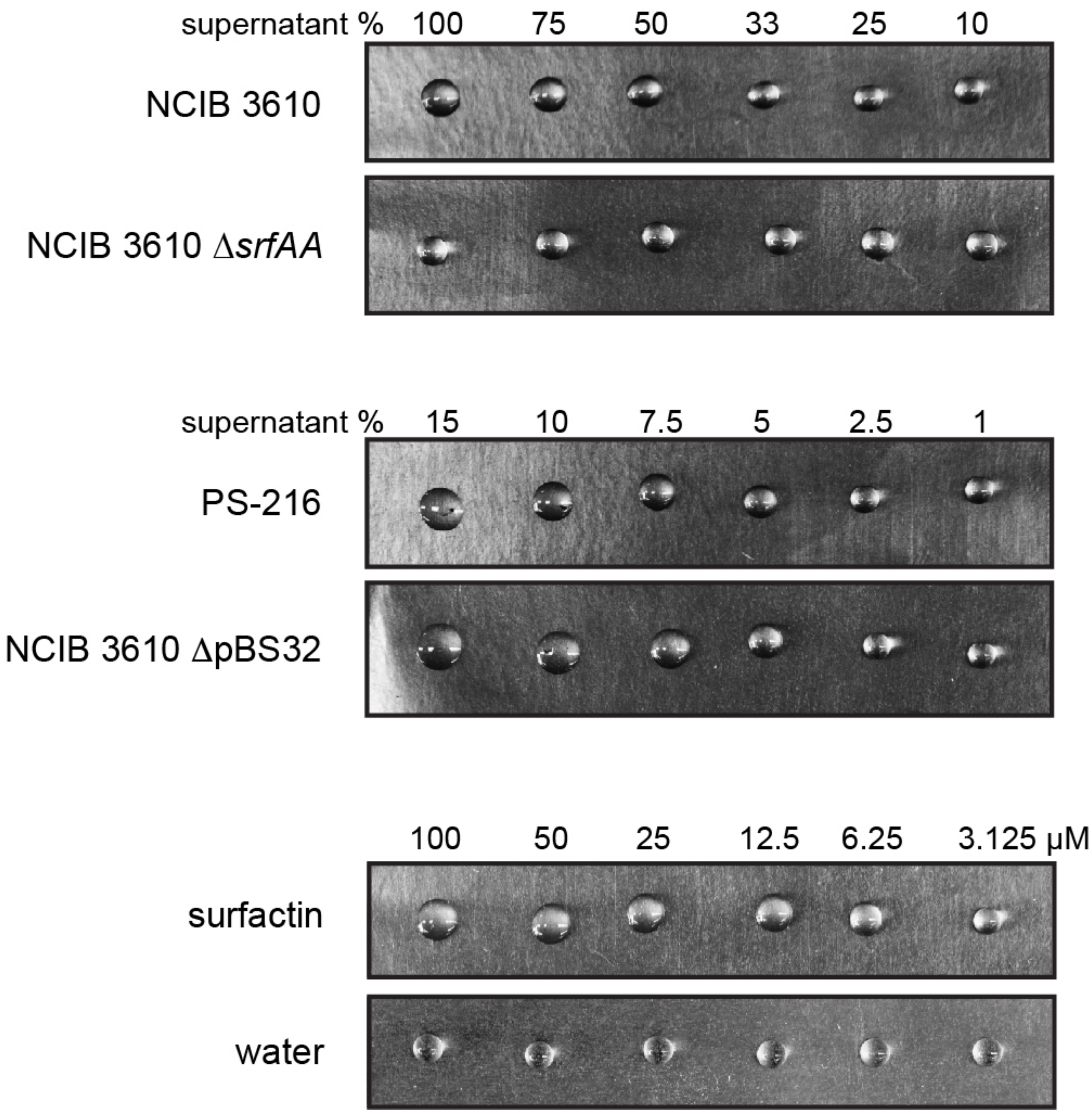

Supplementary Figure 4, related to Table 1. Droplet collapse assay to measure surfactant concentration.

Representative images of swarm supernatants that were serially diluted to the indicated percentages and spotted onto parafilm, then photographed after 10 min and compared to known surfactin concentrations. More collapse of the drops (wider diameters) are indicative of higher amounts of surfactant.

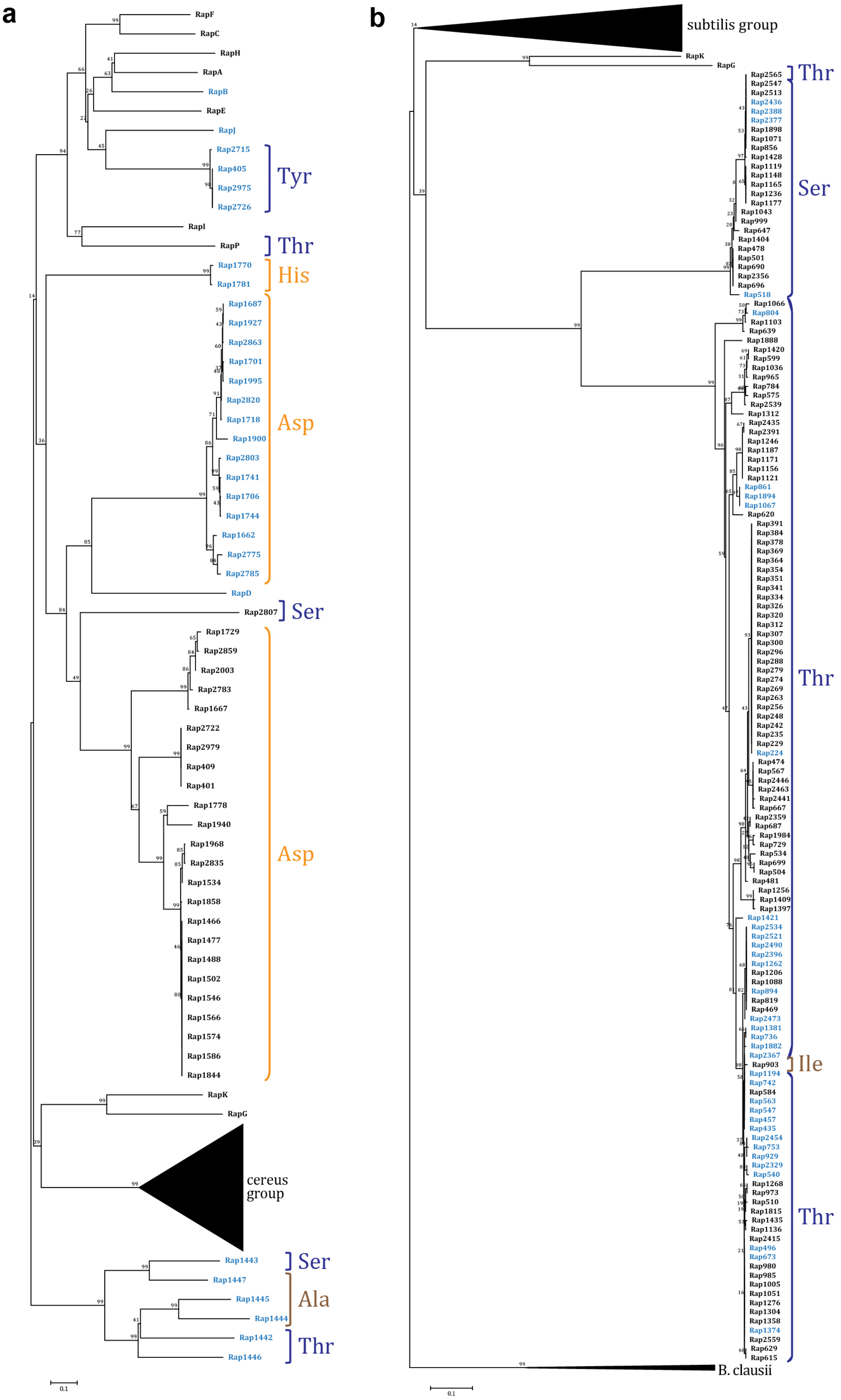
a
RapF
RapC
RapH
RapA
RapB
RapE
RapJ
Rap2715
Rap405
Rap2975
Rap2726
Tyr
RapI
RapP
Thr
Rap1770
Rap1781
His
Rap1687
Rap1927
Rap2863
Rap1701
Rap1995
Rap2820
Rap1718
Rap1900
Rap2803
Rap1741
Rap1706
Rap1744
Rap1662
Rap2775
Rap2785
Asp
RapD
Rap2807
Ser
Rap1729
Rap2859
Rap2003
Rap2783
Rap1667
Rap2722
Rap2979
Rap409
Rap401
Rap1778
Rap1940
Rap1968
Rap2835
Rap1534
Rap1858
Rap1466
Rap1477
Rap1488
Rap1502
Rap1546
Rap1566
Rap1574
Rap1586
Rap1844
Asp
RapK
RapG
cereus group
Rap1443
Ser
Rap1447
Rap1445
Rap1444
Ala
Rap1442
Rap1446
Thr
0.1
b
subtilis group
RapK
RapG
Rap2565
Thr
Rap2547
Rap2513
Rap2436
Rap2388
Rap2377
Rap1898
Rap1071
Rap856
Rap1428
Rap1119
Rap1148
Rap1165
Rap1236
Rap1177
Rap1043
Rap999
Rap647
Rap1404
Rap478
Rap501
Rap690
Rap2356
Rap696
Rap518
Ser
Rap1066
Rap804
Rap1103
Rap639
Rap1888
Rap1420
Rap599
Rap1036
Rap965
Rap784
Rap575
Rap2539
Rap1312
Rap2435
Rap2391
Rap1246
Rap1187
Rap1171
Rap1156
Rap1121
Rap861
Rap1894
Rap1067
Rap620
Rap391
Rap384
Rap378
Rap369
Rap364
Rap354
Rap351
Rap341
Rap334
Rap326
Rap320
Rap312
Rap307
Rap300
Rap296
Rap288
Rap279
Rap274
Rap269
Rap263
Rap256
Rap248
Rap242
Rap235
Rap229
Rap224
Rap474
Rap567
Rap2446
Rap2463
Rap2441
Rap667
Rap2359
Rap687
Rap1984
Rap729
Rap534
Rap699
Rap504
Rap481
Rap1256
Rap1409
Rap1397
Rap1421
Rap2534
Rap2521
Rap2490
Rap2396
Rap1262
Rap1206
Rap1088
Rap894
Rap819
Rap469
Rap2473
Rap1381
Rap736
Rap1882
Rap2367
Rap903
Thr
Ile
Rap1194
Rap742
Rap584
Rap563
Rap547
Rap457
Rap435
Rap2454
Rap753
Rap929
Rap2329
Rap540
Rap1268
Rap973
Rap510
Rap1815
Rap1435
Rap1136
Rap2415
Rap496
Rap673
Rap980
Rap985
Rap1005
Rap1051
Rap1276
Rap1304
Rap1358
Rap1374
Rap2559
Rap629
Rap615
Thr
B. clausii
0.1

Supplementary Figure 5, related to Table 3 and Supplementary Data 2. Phylogenetic tree of all Rap homologs with N236 mutated.

Minimal-evolution tree of all Rap protein sequences with mutations at the equivalent of N236, highlighting either the *subtilis* group and *B. clausii* (A) or the *cereus* group (B); both are from the same tree with different groups compacted for space. The named Raps A-K (with N236 intact) and RapP from *B. subtilis* NCIB 3610 are included for reference. Amino acids listed represent the mutation at position 236, colored by chemical similarity, and orphan Raps with no associated Phr are in blue. Bootstrap values are based on 500 replicates. Based on data from reference 76.

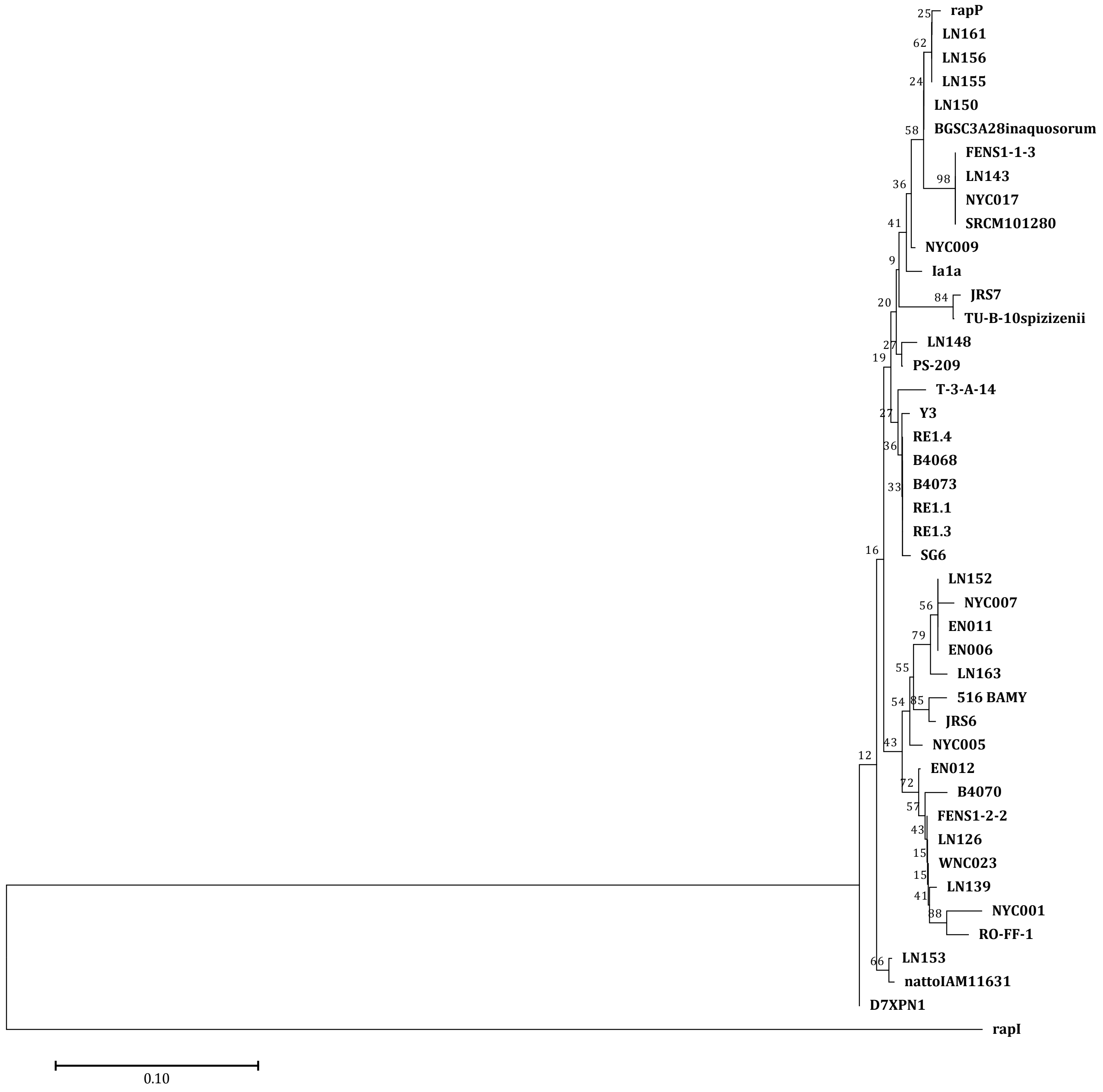

Supplementary Figure 6, related to Table 2 and Supplementary Data 2. Phylogenetic tree of all RapP homologs.

Minimal-evolution tree of all RapP nucleotide sequences found by PCR or BLAST. The nearest Rap homolog *rapI* is included for reference to show that the identified homologs cluster with *rapP*. Bootstrap values are based on 500 replicates.

**Supplementary Table 1. Strains used in experiments in this study.**		
**Number**	**Strain background**	**Genotype**
ZK3814	NCIB 3610	wild type
CY49	NCIB 3610	amyE::Phyperspank-mKate2:cm
NL712	NCIB 3610	amyE::Phyperspank-mKate2:cm, sacA::Pveg-GFP:phleo
NL069	NCIB 3610	srfAA::erm, amyE::Phyperspank-mKate2:cm
NL711	NCIB 3610	srfAA::erm, amyE::Phyperspank-mKate2:cm, sacA::Pveg-GFP:phleo
NL104	NCIB 3610	rapP::Tn10:spc, amyE::Phyperspank-mKate2:cm
NL776	NCIB 3610	rapP::Tn10:spc, amyE::Phyperspank-mKate2:cm, sacA::Pveg-GFP:phleo
NL777	NCIB 3610	rapP::Tn10:spc, srfAA::erm, amyE::Phyperspank-mKate2:cm
NL778	NCIB 3610	rapP::Tn10:spc, srfAA::erm, amyE::Phyperspank-mKate2:cm, sacA::Pveg-GFP:phleo
NL109	NCIB 3610	amyE::PhypercIo3-YFP:spc
NL675	NCIB 3610	srfAA::erm, amyE::PhypercIo3-YFP:spc
NL068	NCIB 3610	hag::tet, amyE::Phyperspank-mKate2:cm
NL699	NCIB 3610	hag::tet, amyE::PhypercIo3-YFP:spc
ZK3664	NCIB 3610	amyE::PsspB-YFP:spc
ZK3666	NCIB 3610	amyE::PtapA-YFP:spc
ZK3776	NCIB 3610	amyE::PsrfAA-YFP:spc
HV1056	NCIB 3610	amyE::PepsA-YFP:spc
ZK3665	NCIB 3610	amyE::Phag-YFP:spc
NL293	PS-216	wild type
NL844	PS-216	lacA::rapPphrP:mls
NL294	PS-216	amyE::Phyperspank-mKate2:cm
NL720	PS-216	amyE::Phyperspank-mKate2:cm, sacA::Pveg-GFP:phleo
NL688	PS-216	srfAA::erm, amyE::Phyperspank-mKate2:cm
NL721	PS-216	srfAA::erm, amyE::Phyperspank-mKate2:cm, sacA::Pveg-GFP:phleo
NL875	PS-216	lacA::rapPphrP:kan, amyE::Phyperspank-mKate2:cm
NL877	PS-216	lacA::rapPphrP:kan, amyE::Phyperspank-mKate2:cm, sacA::Pveg-

		GFP:phleo
NL879	PS-216	srfAA::erm, lacA::rapPphrP:kan, amyE::Phyperspank-mKate2:cm
NL881	PS-216	srfAA::erm, lacA::rapPphrP:kan, amyE::Phyperspank-mKate2:cm, sacA::Pveg-GFP:phleo
NL295	PS-216	amyE::PhypercIo3-YFP:spc
NL687	PS-216	srfAA::erm, amyE::PhypercIo3-YFP:spc
NL832	PS-216	hag::tet, amyE::Phyperspank-mKate2:cm
NL831	PS-216	hag::tet, amyE::PhypercIo3-YFP:spc
NL911	PS-216	amyE::PsrfAA-YFP:spc
NL822	PS-216	amyE::PsspB-YFP:spc
NL791	PS-216	amyE::PepsA-YFP:spc
NL369	PS-216	amyE::PtapA-YFP:spc
NL738	PS-216	amyE::Phag-YFP:spc
NL849	PS-216	lacA::rapPphrP:mls, amyE::PsrfAA-YFP:spc
NL852	PS-216	lacA::rapPphrP:mls, amyE::PsspB-YFP:spc
NL851	PS-216	lacA::rapPphrP:mls, amyE::PepsA-YFP:spc
DS2569	NCIB 3610 ΔpBS32	wild type
NL872	NCIB 3610 ΔpBS32	lacA::rapPphrP:kan
NL905	NCIB 3610 ΔpBS32	lacA::rapP(T236N)phrP:mls
NL034	NCIB 3610 ΔpBS32	amyE::Phyperspank-mKate2:cm
NL726	NCIB 3610 ΔpBS32	amyE::Phyperspank-mKate2:cm, sacA::Pveg-GFP:phleo
NL727	NCIB 3610 ΔpBS32	srfAA::erm, amyE::Phyperspank-mKate2:cm
NL736	NCIB 3610 ΔpBS32	srfAA::erm, amyE::Phyperspank-mKate2:cm, sacA::Pveg-GFP:phleo
NL874	NCIB 3610 ΔpBS32	lacA::rapPphrP:kan, amyE::Phyperspank-mKate2:cm
NL876	NCIB 3610 ΔpBS32	lacA::rapPphrP:kan, amyE::Phyperspank-mKate2:cm, sacA::Pveg-GFP:phleo
NL878	NCIB 3610 ΔpBS32	srfAA::erm, lacA::rapPphrP:kan, amyE::Phyperspank-mKate2:cm
NL880	NCIB 3610 ΔpBS32	srfAA::erm, lacA::rapPphrP:kan, amyE::Phyperspank-mKate2:cm, sacA::Pveg-GFP:phleo
NL907	NCIB 3610 ΔpBS32	lacA::rapP(T236N)phrP:kan, amyE::Phyperspank-mKate2:cm

NL914	NCIB 3610 ΔpBS32	lacA::rapP(T236N)phrP:kan, amyE::Phyperspank-mKate2:cm, sacA::Pveg-GFP:phleo
NL922	NCIB 3610 ΔpBS32	srfAA::erm, lacA::rapP(T236N)phrP:kan, amyE::Phyperspank-mKate2:cm
NL924	NCIB 3610 ΔpBS32	srfAA::erm, lacA::rapP(T236N)phrP:kan, amyE::Phyperspank-mKate2:cm, sacA::Pveg-GFP:phleo
NL926	NCIB 3610 ΔpBS32	amyE::PsrfAA-YFP:spc
NL820	NCIB 3610 ΔpBS32	amyE::PsspB-YFP:spc
NL788	NCIB 3610 ΔpBS32	amyE::PepsA-YFP:spc
NL780	NCIB 3610 ΔpBS32	amyE::PtapA-YFP:spc
NL748	NCIB 3610 ΔpBS32	amyE::Phag-YFP:spc
NL845	NCIB 3610 ΔpBS32	lacA::rapPphrP:mls, amyE::PsrfAA-YFP:spc
NL848	NCIB 3610 ΔpBS32	lacA::rapPphrP:mls, amyE::PsspB-YFP:spc
NL847	NCIB 3610 ΔpBS32	lacA::rapPphrP:mls, amyE::PepsA-YFP:spc
NL916	NCIB 3610 ΔpBS32	lacA::rapP(T236N)phrP:mls, amyE::PsspB-YFP:spc
NL909	NCIB 3610 ΔpBS32	lacA::rapP(T236N)phrP:mls, amyE::PsrfAA-YFP:spc
NL918	NCIB 3610 ΔpBS32	lacA::rapP(T236N)phrP:mls, amyE::PepsA-YFP:spc
