# Supplementary material for: A single mutation in rapP induces cheating to prevent cheating in Bacillus subtilis by minimizing public good production
Source: Commun Biol. 2018 Sep 4;1:133. doi: 10.1038/s42003-018-0136-1 (PMC6123732; doi:10.1038/s42003-018-0136-1)
Supplement: Supplementary file 4 — Description of Additional Supplementary Items [file 42003_2018_136_MOESM4_ESM.docx]

**Supplementary Data 1. Related to Figure 3A. Full gene expression data.**

**Supplementary Data 2. Related to Tables 1 and 2. List of strains examined for *rapP* homologs and N236 mutations.**

Sheet 1 contains the wild strains tested for presence of *rapP* by PCR and the strains with public genomes that contain a *rapP* homolog, plus the sequence of the examined region.

Sheet 2 lists the *Bacillus* species with a N236-mutated Rap protein, along with each Rap ID number, N236 residue, and amino acid sequences of the Rap and Phr proteins.
